# Spatial Scaling of Environmental Variables Improves Species-Habitat Models of Fishes in a Small, Sand-Bed Lowland River

**DOI:** 10.1371/journal.pone.0142813

**Published:** 2015-11-16

**Authors:** Johannes Radinger, Christian Wolter, Jochem Kail

**Affiliations:** 1 Department of Biology and Ecology of Fishes, Leibniz-Institute of Freshwater Ecology and Inland Fisheries, Berlin, Germany; 2 Department of Aquatic Ecology, University of Duisburg-Essen, Essen, Germany; University of Waikato (National Institute of Water and Atmospheric Research), NEW ZEALAND

## Abstract

Habitat suitability and the distinct mobility of species depict fundamental keys for explaining and understanding the distribution of river fishes. In recent years, comprehensive data on river hydromorphology has been mapped at spatial scales down to 100 m, potentially serving high resolution species-habitat models, e.g., for fish. However, the relative importance of specific hydromorphological and in-stream habitat variables and their spatial scales of influence is poorly understood. Applying boosted regression trees, we developed species-habitat models for 13 fish species in a sand-bed lowland river based on river morphological and in-stream habitat data. First, we calculated mean values for the predictor variables in five distance classes (from the sampling site up to 4000 m up- and downstream) to identify the spatial scale that best predicts the presence of fish species. Second, we compared the suitability of measured variables and assessment scores related to natural reference conditions. Third, we identified variables which best explained the presence of fish species. The mean model quality (AUC = 0.78, area under the receiver operating characteristic curve) significantly increased when information on the habitat conditions up- and downstream of a sampling site (maximum AUC at 2500 m distance class, +0.049) and topological variables (e.g., stream order) were included (AUC = +0.014). Both measured and assessed variables were similarly well suited to predict species’ presence. Stream order variables and measured cross section features (e.g., width, depth, velocity) were best-suited predictors. In addition, measured channel-bed characteristics (e.g., substrate types) and assessed longitudinal channel features (e.g., naturalness of river planform) were also good predictors. These findings demonstrate (i) the applicability of high resolution river morphological and instream-habitat data (measured and assessed variables) to predict fish presence, (ii) the importance of considering habitat at spatial scales larger than the sampling site, and (iii) that the importance of (river morphological) habitat characteristics differs depending on the spatial scale.

## Introduction

Species distribution or habitat models are widely used in conservation planning and the management of natural systems to: (i) statistically analyse species’ ecological needs based on empirical data from a number of sampling sites, and (ii) predict species’ presence for large areas based on species-habitat relationships. The environmental predictor variables must be available for the whole area of interest, and thus, such models are usually based on areal data like climate, geology, and elevation [1].

In recent years, inclusive and comprehensive data on river hydromorphology covering whole river networks have been mapped and compiled to describe the habitat conditions for e.g. national watershed management and monitoring programs (reviewed within the project REFORM [2]). In general, hydromorphology links hydrology and geomorphology and incorporates the hydrological regime, river morphology, and river continuity [2]. In particular, hydromorphological assessments of the physical habitats (e.g. the German method of the Länderarbeitsgemeinschaft Wasser (LAWA) [3]) typically map channel dimensions, indicators for morphodynamics, channel-bed and bank features, substrates and the structure of the riparian zone. These data can be used to investigate and model the distribution of stream biota such as fish in relation to the hydromorphological habitat conditions. However, there are several particularities of the hydromorphological data which have to be considered:

First, the hydromorphological data usually have a high spatial resolution, e.g. 100 m (Germany: LAWA, [3]) and 500 m (Britain and Ireland: River Habitat Survey, [4]) compared to the high mobility of riverine fish [5,6]. Fish generally undertake various movements ranging from small-scale home-range movements (e.g. diel movements within and between habitats associated with foraging or avoidance of predators) to large-scale life-cycle related migrations (e.g. spawning runs) and non-migratory dispersal [7]. Therefore, besides the local habitat conditions, the hydromorphological characteristics up- and downstream of a sampling site potentially influence and might be good predictors of fish species’ presence [8]. Hence, larger spatial scales should be considered in modelling species-habitat relationships. Besides increasing the predictive power of the models, such species-habitat relationships at larger spatial scales might reflect or approximate the minimum spatial extent of a specific habitat required that contains suitable spawning substrates, littoral nurseries for larvae and juveniles, as well as feeding grounds and overwintering habitats for all age groups of a species. This is of special importance in river rehabilitation, e.g. for planning and dimensioning stepping stone habitats or the necessary spatial extent of successful restoration measures.

Second, hydromorphological datasets are provided as two fundamentally different types of variables [9]: i) Measured variables that are obtained in the field and quantify specific habitat characteristics (e.g. channel sinuosity, dominant substrate), and ii) assessment scores which describe the deviation of the measured variable from stream-type specific natural reference conditions and usually range from unchanged (only minor deviations from the reference conditions) to heavily degraded. Assessment scores relate the measured variables to the natural conditions to which fish have adapted. Thus, the usefulness of assessment scores for species-habitat models strongly depends on (i) the knowledge on the specific habitat needs of fish and (ii) the definition of natural reference conditions and naturalness, which is at least partly subjective, and if these two aspects have been adequately considered in the assessment scores. Moreover, it is of ecological interest to identify the hydromorphological variables, which best explain the presence of fish species. Even though many hydromorphological variables are co-correlated [10], and hence, it is difficult to directly infer causal relationships, such results would indicate which hydromorphological habitat conditions are of special importance in river management and restoration.

We are not aware of any study that used high resolution hydromorphological data to develop species-habitat models and i) considers fish mobility to identify spatial scales which best explain fish species presence and ii) explicitly investigates the predictive power of measured compared to assessed environmental variables. This study has used a spatially inclusive and comprehensive dataset on river hydromorphology of a lowland sand-bed river in northern Germany to develop species-habitat models for 13 fish species. The first objective was to test if habitat conditions at larger spatial scales, i.e. up- and downstream reaches adjacent to the sampling sites, improve the predictive power of fish-habitat models. It was hypothesized that the predictive power increases up to a certain spatial distance. The second objective was to compare the predictive power of the two main types of hydromorphological variables: quantitative, measured variables and assessment scores. It was hypothesized that the measured variables yield better modelling results since they relate the presence of a species to the directly perceived physical environment, while assessment scores might be affected by the subjective definition of naturalness. The third objective was to identify the hydromorphological variables at different spatial scales that are related best to the presence of fish species.

## Methods

### Study river catchment

**Fig 1:**
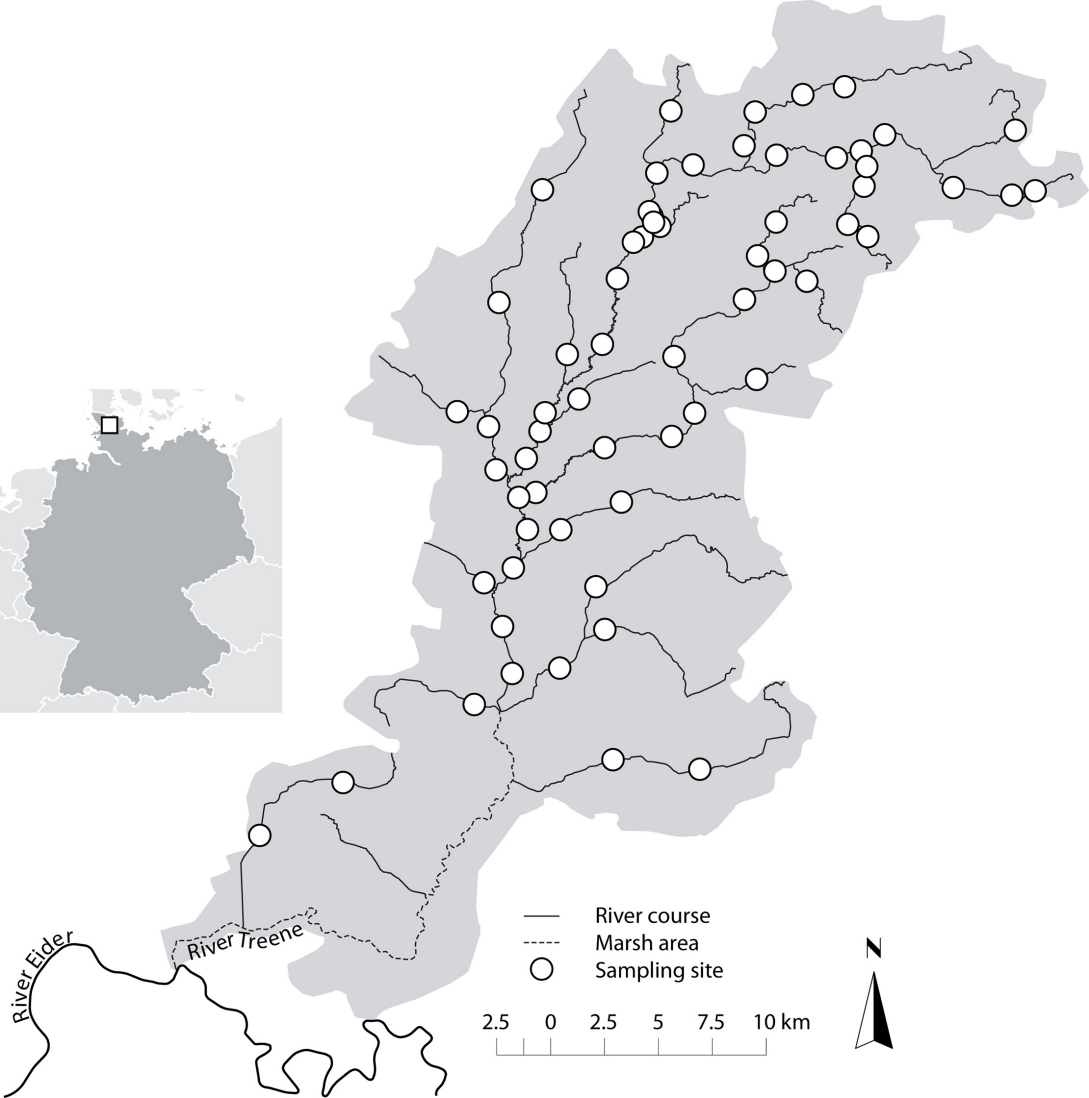
Overview of the River Treene catchment (760 km^2^, Germany) and 64 sampling sites.

The study area is located in northern Germany and comprises the whole catchment (760 km^2^) of the River Treene (Fig 1, bounding box N: 54°46'19''N, S: 54°21'36''N, W: 9°04'50''E, E: 9°44'01''E). The 77 km long River Treene is naturally meandering, mainly sand-dominated with local gravel patches and low valley slopes in a highly agriculturally dominated catchment (89% agriculture, CORINE Land Cover 2006). Most of the upstream river network are small gravel-dominated lowland streams (LAWA river type 16, [11]) belonging to the hyporhithral region according to Illies [12] with *Leuciscus leuciscus*, *Phoxinus phoxinus* and *Salmo trutta* as key fish species. The middle, epipotamal reaches are small to large sand and loam-dominated lowland rivers (LAWA river type 14 and 15) typically dominated by e.g. *Barbatula barbatula*, *Gobio gobio*, *Leuciscus leuciscus* and *Gasterosteus aculeatus*. The downstream marshland streams of the coastal plains (LAWA river type 22) are tidally influenced and belong to the metapotamal with key fish species like *Rutilus rutilus*, *Perca fluviatilis* and *Abramis brama* [13,14]. Large parts of the river have been straightened, 66% of its length is in a poor or bad hydromorphological state and the river network is fragmented by a total of 52 barriers (0.16/km) (S1 Fig). However, a few short, near-natural meandering reaches are still present (1.5% of the river length). The marshy downstream sections were excluded from the analysis, because of both, the river morphology and ecology strongly differed from the rest of the catchment and no hydromorphological data were available.

### Species data

All fish abundance data were sampled at 64 sites in 2004–2011 by the State Agency for Agriculture, Environment and Rural Areas (LLUR) of the federal state of Schleswig Holstein, and kindly provided for the study. These samples were collected using electric fishing along river stretches of on average 400 m length (160–1100 m) following the recommendations given by the national fish-based assessment system [15]. For each sampling site, species data of repeated sampling over multiple years were pooled to account for inter-annual variations, and abundance data were converted to presence-absence data for each species. All fish species present (at least one specimen) at ≥ 10 sampling sites were selected for this study, resulting in 13 model species (Table 1).

**Table 1:** Fish species included in the analysis and their presence, absence and occurrence frequency in 64 sampled sites.

Code	Common name	Scientific name	Presence	Absence	Occurrence frequency
Anguilla	European eel	*Anguilla anguilla*	48	16	0.75
Cobienia	Spined loach	*Cobitis taenia*	20	44	0.31
Gastatus	Three-spined stickleback	*Gasterosteus aculeatus*	54	10	0.84
Gobiobio	Gudgeon	*Gobio gobio*	46	18	0.72
Gymnrnua	Ruffe	*Gymnocephalus cernua*	10	54	0.16
Leucscus	Common dace	*Leuciscus leuciscus*	27	37	0.42
Percilis	European perch	*Perca fluviatilis*	37	27	0.58
Phoxinus	Eurasian minnow	*Phoxinus phoxinus*	29	35	0.45
Pungtius	Nine-spined stickleback	*Pungitius pungitius*	48	16	0.75
Rutiilus	Roach	*Rutilus rutilus*	33	31	0.52
Salmalar	Atlantic salmon	*Salmo salar*	19	45	0.30
Salmario	Brown trout	*Salmo trutta*	48	16	0.75
Tincinca	Tench	*Tinca tinca*	11	53	0.17

### Environmental data

The environmental dataset comprised 35 measured hydromorphological habitat variables, 13 hydromorphological assessment variables, and has been complemented by 3 topological variables (i.e. stream order typology and distance from mouth) (Table 2). The hydromorphological data were provided by LLUR and were recorded for homogenous channel segments of generally 100 m length using a method that closely refers to the standard assessment method of the LAWA [16] described by Gellert et al. [3].

**Table 2:** Environmental variables used in the analysis, assigned main variables groups and corresponding descriptive values. Asterisks indicate aggregated variables derived by pooling multiple subcategories (e.g. %mud, %clay and %silt was summed up to %SuSo, soft substrates).

Code	Group	Variable	Mean (Standard Deviation)
**Topological Variables**			
DisM	TOPO	Distance from mouth (m)	51181.13 (18354.64)
SOSh	TOPO	Stream order according to Shreve (1966)	2.55 (3.34)
SOSt	TOPO	Stream order according to Strahler (1957)	1.38 (0.59)
**Measured Variables**			
ChDe	PROFILE	Channel depth (m)	0.45 (0.38)
ChWi	PROFILE	Channel width (m)	3.98 (4.53)
ChWV	PROFILE	Channel width variability categories of 1: no, 2: low, 3: medium, 4: high, 5: very high	1.73 (0.68)
CSFo	PROFILE	Cross-section form categories of 1: natural, 2: near natural, 3: erosive cross-section—varying, 4: failed embankment, 5: erosive cross-section–deep, 6: trapezoid, 7: V-shaped, 8: rectangular	5.12 (1.99)
FlVe	PROFILE	Flow velocity categories of 1: no (<5 cms^-2^), 2: low (5–20 cms^-2^), 3: medium (20–40 cms^-2^), 4: high (40–80 cms^-2^), 5:very high (>80 cms^-2^)	2.81 (0.87)
BAEr*	BED	Bed alteration–erosion, moving sands (n/100 m)	0.11 (0.32)
BAOt*	BED	Bed alteration–others (e.g. clogging, unnamed categories) (n/100 m)	0.09 (0.36)
BAWa*	BED	Bed alteration–waste deposition (n/100 m)	0.12 (0.41)
CBFO*	BED	Channel bed features–others (e.g. cascades, unnamed categories) (n/100 m)	0.06 (0.53)
CBFR*	BED	Channel bed features–riffles, pools (n/100 m)	0.06 (0.31)
InVe	BED	Instream vegetation categories of 1: no, 2: submerged, 3: floating leaved, 4: emerged macrophytes	1.82 (0.96)
SMaS	BED	Submerged macrophyte species (n)	0.74 (0.9)
SuDi	BED	Substrate diversity categories of 1: no, 2: low, 3: medium, 4: high, 5: very high	0.85 (0.33)
SuHa*	BED	Substrate–hard (e.g. gravel, stones) (%)	14.68 (22.45)
SuMa	BED	Substrate–macrophytes (%)	4.17 (7.45)
SuSa	BED	Substrate–sand (%)	57.74 (25.88)
SuSo*	BED	Substrate–soft (e.g. mud, clay, silt) (%)	21.29 (23.2)
SuWo*	BED	Substrate—wood (e.g. dead wood, rootstock) (%)	2.12 (8.08)
BFLW*	BANK	Bank features–large wood (n/100 m)	0.06 (0.32)
BFOt*	BANK	Bank features–others (e.g. nesting bank) (n/100 m)	0.04 (0.23)
BPGr*	BANK	Bank protection–green categories of 0: no, 1: one bank, 2: both banks	0.02 (0.17)
BPWa	BANK	Bank protection–walls categories of 0: no, 1: one bank, 2: both banks	0.01 (0.11)
BPno*	BANK	no Bank protection categories of 0: no, 1: one bank, 2: both banks	1.45 (0.87)
BPRi	BANK	Bank protection–riprap categories of 0: no, 1: one bank, 2: both banks	0.03 (0.22)
BPWo*	BANK	Bank protection–wood categories of 0: no, 1: one bank, 2: both banks	0.44 (0.8)
RVRe	BANK	Riparian vegetation–reeds categories of 0: no, 1: one bank, 2: both banks	0.03 (0.18)
RVSp*	BANK	Riparian vegetation–sparse categories of 0: no, 1: one bank, 2: both banks	1.73 (0.56)
RVTF*	BANK	Riparian vegetation–trees, forest categories of 0: no, 1: one bank, 2: both banks	0.24 (0.54)
CFIB*	LONG	Channel features–islands braiding (n/100 m)	0.02 (0.16)
CFLW*	LONG	Channel features–large wood (n/100 m)	0.02 (0.22)
CFNa	LONG	Channel features–narrowing (n/100 m)	0.09 (0.36)
CFWi	LONG	Channel features–widening (n/100 m)	0.09 (0.45)
ChDV	LONG	Channel depth variability categories of 1: no, 2: low, 3: medium, 4: high, 5: very high	1.61 (0.66)
FlDi	LONG	Flow diversity categories of 1: no, 2: low, 3: medium, 4: high, 5: very high	1.77 (0.61)
Plan	LONG	Planform categories of 1: heavily meandering, 2: meandering, 3: strongly sinuous, 4: sinuous, 5: slightly sinuous, 6: straight, 7: channelized	5.65 (1.36)
**Assessment Variables (functional units)**			
FE-CSD	PROFILE	Cross section depth (score)	4.48 (0.97)
FE-CSF	PROFILE	Cross section form (score)	3.89 (1.22)
FE-CSW	PROFILE	Cross section width (score)	3.44 (1.1)
FE-BeF	BED	Bed fixation (score)	2.02 (0.16)
FE-Sub	BED	Substrate (score)	4.21 (0.66)
FE-BaP	BANK	Bank protection (score)	2.34 (0.74)
FE-BFe	BANK	Bank features (score)	4.79 (0.64)
FE-RVe	BANK	Riparian vegetation (score)	3.63 (0.63)
FE-ChD	LONG	Channel dynamic (score)	3.66 (0.72)
FE-LPr	LONG	Longitudinal profile (score)	4.48 (0.49)
FE-Pla	LONG	Planform (score)	4.57 (0.62)
FE-FPl	FLOODPLAIN	Floodplain (score)	3.1 (0.57)
FE-RBS	FLOODPLAIN	Riparian buffer strip (score)	4.09 (1.19)

The 35 measured and 13 assessment variables were grouped according to the aspect of river hydromorphology they describe based on the LAWA on site survey [3]: channel cross-profile (PROFILE, e.g. flow velocity, depth and width), channel bed (BED, e.g. substrates, number of channel features like riffles and pools), channel banks (BANK, e.g. bank fixation), channel planform (LONG, e.g. sinuosity), adjacent floodplain (FLOODPLAIN, only 2 assessment variables, Table 2). For the 13 assessment variables, the deviation from stream-type specific natural reference conditions was assessed by trained LLUR experts on a five-point ordinal scale ranging from undisturbed (1) to heavily degraded (5) following a standardized procedure [17,18]. The topological variables used were distance from mouth (i.e. from the confluence with River Eider) and stream order according to Strahler [19] and Shreve [20]. Both methods, Strahler and Shreve assign headwater streams an order of one and increase in downstream direction. The commonly used Strahler order [19] only increases if at least two tributary branches of the same Strahler order meet (i.e. two second-order streams form a third order stream). Streams of lower order joining a higher order stream do not affect the Strahler order at the confluence. In contrast, the Shreve order [20] equals the sum of the Shreve orders of the tributaries, and hence, is often considered a better proxy of the size of the river (width or discharge), especially in elongated river networks with a high number of low order tributaries.

For the analysis, some of the originally measured variables were thematically pooled into aggregated variables if their individual effects on fish could be expected the same. The aggregated variables were calculated as sums of subcategories (e.g. % of soft substrates is the sum of %mud, %clay and %silt) (Table 2). For variables that represent counts, values were standardized by the length of the corresponding mapped river segment. The vector data were converted into raster data by rasterizing the river network with a model grid cell size of 50 x 50 m using the GRASS GIS tool *v*.*to*.*rast*.

Species’ mobility was accounted for by summarizing the habitat conditions in up- and downstream reaches and calculating average habitat values at four predefined distances (200, 1000, 2500, 4000 m) covering a range of movement and home-range distances from smaller and more stationary fish species to larger and more mobile fish species [5]. We used a GIS-based neighbourhood focal filter tool (GRASS GIS, *r*.*rdfilter*). This focal filter (also referred to as low-pass filter) is a spatial averaging filter that smooths the data by reducing variation in the neighbourhood. In fact, parameter values of adjacent cells in a given distance are averaged and the calculated mean or median value is assigned to the focal centre cell. Consequently, each cell contains an average value of a respective parameter over a defined neighbourhood distance. For this analysis, all model grid cells in the four predefined distances up- and downstream of the fish sampling sites were used to calculate mean and median predictors for continuous and ordinal variables respectively, referred to as distance classes in the following. Consequently, the total length of the considered reach is twice the distance, e.g. 5000 m for the 2500 m distance class. The calculation of distance-dependent predictors included also tributaries, but was principally limited to grid cells up- or downstream to the next impassable barrier. Finally, in addition to the habitat characteristics at the level of the site (referred to as 0 m distance class), average values of the four new distance classes were calculated for a total of 51 variables (35 measured, 13 assessed, 3 topological). Subsequently, different sets of variables were assembled; including either measured or assessed variables, one out of the five spatial scales, and with and without topological variables, resulting in 2*5*2 = 20 environmental predictor datasets (Table 2).

### Modelling and statistical analysis

Boosted regression trees (BRT) were built for each of the 20 predictor-datasets and each of the 13 selected fish species to examine the relationship between the environmental variables and species occurrence. BRT is a statistical learning method that additively combines and averages (boosting) many simple single regression trees to form a collective model of improved predictive performance [21,22]. Moreover, BRTs can accommodate continuous and categorical variables, are not affected by missing values or transformation or outliers and are considered to effectively select relevant variables, identify variable interactions and thus avoid overfitting [23]. Species-habitat relationships were modelled based on a binary response (presence and absence records, Bernoulli distribution) in a three-step analysis framework in the statistical software R (specifically the R-package ‘dismo’ [24]) following general guidelines on BRTs proposed by Elith and Leathwick [25]: First, for each species a global model was built including all variables per dataset. Second, the model was simplified and the predictor variable set was reduced by integrated backward elimination of variables that gave no evidence of improving the model. Third, the final model was calculated based on the reduced predictor variable datasets. For each of the global and final BRT models, the tree complexity and learning rate was set to 3 respectively 0.001 or smaller to achieve the recommended number of more than 1000 regression trees [23]. All other model settings were set to default or were automatically adjusted by the boosting algorithm. The small number of positive occurrences of some species did not allow splitting the dataset for extensive model evaluations. Instead, a commonly used 10-fold cross validation already implemented in the algorithm was carried out. As a measure of the model’s predictive quality, the mean AUC (area under the receiver operating characteristic (ROC) curve), a fold-based statistic using the cross-validated model, was extracted. The AUC is a threshold-independent rank-correlation coefficient where high values typically indicate a strong agreement between the predicted suitability and known presences and absences, respectively [26]. Here, the AUC is calculated model internally (R-function ‘gbm.step{dismo}’) based on a non-parametric rank-correlation closely related to the Mann-Whitney *U* statistic [27]:

$$AUC\;=\;\frac{U}{n_{1}n_{2}}$$ (1)

where *U* = *U*-statistic according to Mann and Whitney [28] of the observed presences/absences (binary) and the predicted suitability (probability), n_1_ = number of observed presences, n_2_ = number of observed absences. Thus, in contrast to other quality measures of predictive performance (e.g. kappa statistic [29]), this method does not rely on the essentially arbitrary choice of a threshold probability to discriminate between absences and presences [30]. It is rather the predicted probabilistic suitability that is evaluated against the observed occurrence patterns. Moreover, the AUC can be used to classify model performance based on five classes (e.g. [31]): <0.6 (fail), 0.6–0.7 (poor), 0.7–0.8 (fair), 0.8–0.9 (good), >0.9 (excellent).

Results of the importance of the single variables were assembled and presented in six main variable groups (TOPO, PROFILE, BED, BANK, LONG, FLOODPLAIN, Table 2, column ‘Group’). In addition to the absolute variable selection frequency of each group and to allow comparisons between groups comprising different numbers of variables, variable selection frequency was standardized to frequency per model and variable (standardized selection frequency, SSF). Furthermore, the relative importance (%) of each predictor variable in the final BRT models was quantified based on the number of times a variable was used for splitting, weighted by the squared improvement at each split and averaged over all trees [23]. Differences in the importance of single predictor variables groups were analysed with a Kruskal-Wallis test (R-function ‘kruskal.test{stats}’, [32]) followed by pairwise Wilcoxon Rank Sum tests with Bonferroni correction (R-function ‘pairwise.wilcox.test{stats}’, [28,33]).

To evaluate and quantify the single and joint effects of (i) the spatial scale over five distance classes (iii) the environmental variable set (measured vs. assessed variables) and (iii) the inclusion of topological information (with vs. without topological information), on the cross-validation AUC, a linear mixed model [34] was fitted. Species was included as random effect to account for potential differences in species-specific habitat associations and differences in spatial movement abilities and home-range extent among the species [5]. Parametric bootstrapping was applied to obtain the model coefficients’ 95% confidence intervals (CI, 1000 simulations, percentile method, [35]). Additionally, for testing for significant differences between model pairs (e.g. models with and without topological information) paired Welch’s t-tests were calculated. For all statistical analysis, the cross-validation AUC (response), which is ranging between zero and one, was arcsine-square root transformed for meeting assumptions for parametric statistical tests.

All spatial analysis were carried out in GRASS GIS (version 6.5SVN, [36]) using its standard functionalities (e.g. *r*.*mapcalc*) and the add-on *r*.*rdfilter* for calculating distance dependent predictor variables. Statistical analyses were carried out in R (version 3.0.1, [37]) using built-in functions from the *base* package, the gbm-functions from the package *dismo* (version 0.8–11, [24]) for BRT model building, *spgrass6* (version 0.8–1, [38]) and *raster* (version 2.1–25, [39]) for the interaction with GRASS GIS and *lme4* (version 1.1–1, [40]) for fitting linear mixed models.

## Results

Overall, model performance was good and sufficient to predict the presence of all 13 model fish species, with a mean cross-validated AUC over all models of 0.782 (SD = 0.092) (Table 3). The modelling algorithm did not converge and failed to compute two models (*T*. *tinca–*measured variables, distance 2500 m; *G*. *aculeatus*–assessed variables, distance 0 m), that were excluded from further analysis.

**Table 3:** Summary of model performances (cross-validation AUC) for models with (A) topological variables excluded and (B) topological variables included contrasting 13 modelled species (for abbreviations see Table 1), five distance classes and two variable datasets (MV: measured variables, AV: assessment scores).

		0 m		200 m		1000 m		2500 m		4000 m				
(A)	Species	MV	AV	MV	AV	MV	AV	MV	AV	MV	AV	mean MV	mean AV	mean
	Anguilla	0.61	0.68	0.63	0.75	0.81	0.83	0.76	0.82	0.73	0.82	**0.71**	**0.78**	**0.74**
	Cobienia	0.65	0.75	0.69	0.69	0.69	0.78	0.66	0.82	0.78	0.79	**0.69**	**0.77**	**0.73**
	Gastatus	0.72		0.78	0.70	0.81	0.73	0.75	0.90	0.76	0.83	**0.77**	**0.79**	**0.78**
	Gobiobio	0.94	0.75	0.94	0.74	0.96	0.90	0.97	0.84	0.88	0.86	**0.94**	**0.82**	**0.88**
	Gymnrnua	0.68	0.79	0.72	0.83	0.69	0.72	0.78	0.82	0.92	0.75	**0.76**	**0.78**	**0.77**
	Leucscus	0.78	0.72	0.68	0.75	0.83	0.73	0.75	0.75	0.65	0.76	**0.74**	**0.74**	**0.74**
	Percilis	0.80	0.80	0.90	0.83	0.78	0.76	0.93	0.85	0.85	0.88	**0.85**	**0.82**	**0.84**
	Phoxinus	0.73	0.74	0.83	0.88	0.89	0.83	0.87	0.91	0.88	0.84	**0.84**	**0.84**	**0.84**
	Pungtius	0.72	0.71	0.69	0.77	0.72	0.68	0.67	0.78	0.62	0.74	**0.68**	**0.73**	**0.71**
	Rutiilus	0.69	0.72	0.69	0.75	0.70	0.66	0.74	0.71	0.73	0.67	**0.71**	**0.70**	**0.71**
	Salmalar	0.88	0.85	0.87	0.83	0.92	0.92	0.88	0.89	0.92	0.89	**0.89**	**0.88**	**0.89**
	Salmario	0.74	0.70	0.83	0.65	0.90	0.71	0.76	0.54	0.88	0.66	**0.82**	**0.65**	**0.74**
	Tincinca	0.76	0.72	0.67	0.61	0.53	0.86		0.75	0.78	0.75	**0.69**	**0.74**	**0.71**
	**mean**	**0.75**	**0.74**	**0.76**	**0.75**	**0.79**	**0.78**	**0.79**	**0.80**	**0.80**	**0.79**	**0.78**	**0.77**	**0.77**
**(B)**	Anguilla	0.72	0.81	0.66	0.80	0.73	0.87	0.77	0.90	0.87	0.76	**0.75**	**0.83**	**0.79**
	Cobienia	0.69	0.77	0.70	0.62	0.77	0.76	0.79	0.79	0.73	0.84	**0.73**	**0.75**	**0.74**
	Gastatus	0.84	0.78	0.70	0.81	0.61	0.84	0.68	0.92	0.71	0.83	**0.71**	**0.83**	**0.77**
	Gobiobio	0.91	0.73	0.92	0.85	0.92	0.88	0.97	0.84	0.89	0.86	**0.92**	**0.83**	**0.88**
	Gymnrnua	0.69	0.77	0.77	0.79	0.73	0.68	0.83	0.75	0.80	0.75	**0.76**	**0.75**	**0.75**
	Leucscus	0.79	0.78	0.73	0.79	0.81	0.74	0.80	0.68	0.77	0.79	**0.78**	**0.76**	**0.77**
	Percilis	0.85	0.85	0.89	0.83	0.80	0.87	0.78	0.89	0.86	0.83	**0.84**	**0.86**	**0.85**
	Phoxinus	0.82	0.92	0.83	0.87	0.83	0.83	0.88	0.90	0.94	0.88	**0.86**	**0.88**	**0.87**
	Pungtius	0.68	0.64	0.58	0.73	0.80	0.76	0.67	0.71	0.67	0.74	**0.68**	**0.71**	**0.70**
	Rutiilus	0.75	0.77	0.78	0.76	0.72	0.74	0.81	0.80	0.78	0.81	**0.77**	**0.78**	**0.77**
	Salmalar	0.97	0.93	0.98	0.93	0.98	0.92	0.96	0.95	0.91	0.92	**0.96**	**0.93**	**0.94**
	Salmario	0.82	0.77	0.77	0.74	0.80	0.86	0.80	0.69	0.76	0.62	**0.79**	**0.74**	**0.76**
	Tincinca	0.58	0.63	0.67	0.65	0.49	0.78	0.62	0.72	0.75	0.72	**0.62**	**0.70**	**0.66**
	**mean**	**0.78**	**0.78**	**0.77**	**0.78**	**0.77**	**0.81**	**0.80**	**0.81**	**0.80**	**0.80**	**0.78**	**0.80**	**0.79**

**Fig 2:**
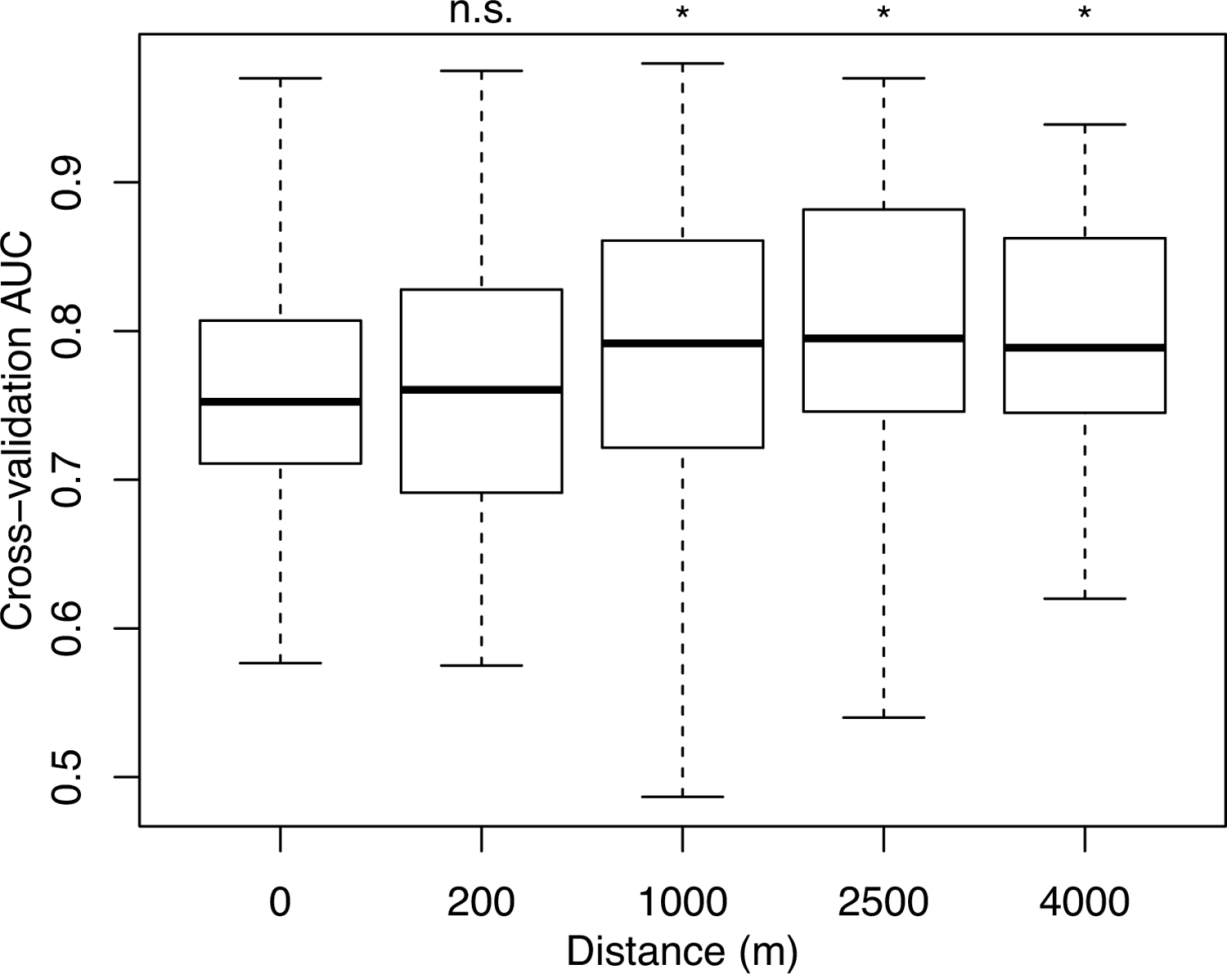
Cross-validated model performances for all species and across five distance classes. Model performance (AUC, area under the receiver operating characteristic curve) increases with distance and significant effects were detected for 1000 m (linear mixed model, β_D1000_ = 0.041, 95%-CI = 0.009–0.075), 2500 m (β_D2500_ = 0.049, 95%-CI = 0.017–0.080) and 4000 m (β_D4000_ = 0.040, 95%-CI = 0.011–0.072).

The models’ predictive power (cross-validated AUC) progressively increased when information on the hydromorphological habitat conditions at longer reaches up- and downstream up to a distance of 2500 m was included (Fig 2). The linear mixed model with species as random effects (no interactions) revealed that the model’s AUC for the three modelled distance classes 1000, 2500 and 4000 m were significantly higher than those of the 0 m distance class (Table 4). The AUC values of the models with habitat conditions aggregated over 2500 m up- and downstream (columns 7 and 8 in Table 3) showed the largest significantly positive differences (β_D2500_ = 0.049, CI = 0.017–0.080) compared to a base model using the local conditions only (0 m distance class), followed by the distance classes 1000 m (β_D1000_ = 0.041, CI = 0.009–0.075) and 4000 m (β_D4000_ = 0.040, CI = 0.011–0.072). In contrast, the effect of the shortest modelled distance class of 200 m (β_D200_ = 0.012, CI = -0.018–0.046) was not significantly different from the 0 m distance class. Species-habitat models performed best for the 2500 m–distance class and with topological variables included.

**Table 4:** Results of the linear mixed effects model. Fixed effect size estimates of (i) four distance classes (200, 1000, 2500 and 4000 m), (ii) the inclusion of topological variables (TV) and (iii) the choice of the environmental dataset (measured MV vs. assessed AV). Effect sizes are estimates how the cross-validated AUC changes compared to the base model for measured variables without TV at distance class 0.The linear mixed model structure follows: *arcsin(√ AUC) ~ α+β*_*D*_ *+ β*_*TV*_ *+ β*_*AV*_ *+ a*_*Species*_, where *α* = intercept, *β* = single effect sizes and *a*_*Species*_ = within species as random effect. 95%-Confidence intervals (CI) are based on parametric bootstrapping. Significant effects are highlighted in bold.

	Parameter estimate	95% CI
*α*	**1.07**	**1.01–1.13**
*β*_*D200*_	0.012	-0.018–0.046
*β*_*D1000*_	**0.041**	**0.009–0.075**
*β*_*D2500*_	**0.049**	**0.017–0.080**
*β*_*D4000*_	**0.040**	**0.011–0.072**
*β*_*TV*_	**0.029**	**0.009–0.049**
*β*_*AV*_	-0.015	-0.036–0.005
*a*_*Species*_	0.092	0.051–0.132

The predictive power of models which were based on measured variables was not significantly different from models based on assessment variables (t-test, t_127_ = 0.10, p = 0.917, two-tailed). The mean AUC of models based on assessed variables was 0.784 (n = 129, SD = 0.081, IQR = 0.733–0.840, columns 2, 4, 6, 8, 10 in Table 3); the mean AUC of models based on measured variables was 0.780 (n = 129, SD = 0.102, IQR = 0.703–0.858, columns 1, 3, 5, 7, 9 in Table 3). For five out of the 13 modelled species, significant differences between the performance of models based on measured and assessed variables were found: two species were more accurately modelled using assessed variables (two-sided Wilcoxon signed rank test, *A*. *anguilla*: p = 0.032; *G*. *aculeatus*: p = 0.098); three other species using measured variables (two-sided Wilcoxon signed rank test, *G*. *gobio*: p = 0.002; *S*. *salar*: p = 0.044; *S*. *trutta*: p = 0.019). Furthermore, variation in the AUC values was significantly lower in the dataset with assessed variables (F-test, F_128,128_ = 1.73, p = 0.001, one-tailed).

Mean overall model performance significantly improved from 0.775 to 0.789 (t_127_ = 2.59, p = 0.011, two-tailed) when topological predictor variables were included. The linear mixed model with species as random effects revealed a significant main effect for the inclusion of topology β_TV_ = 0.029 (CI = 0.009–0.049, Table 4). Moreover, a Spearman rank correlation analysis indicated a positive correlation with fish length (rho = 0.24, p = 0.006), i.e. the model improvement due to including topological variables was higher for larger species (e.g. diadromous *A*. *anguilla* and *S*. *salar*) than for smaller species.

**Fig 3:**
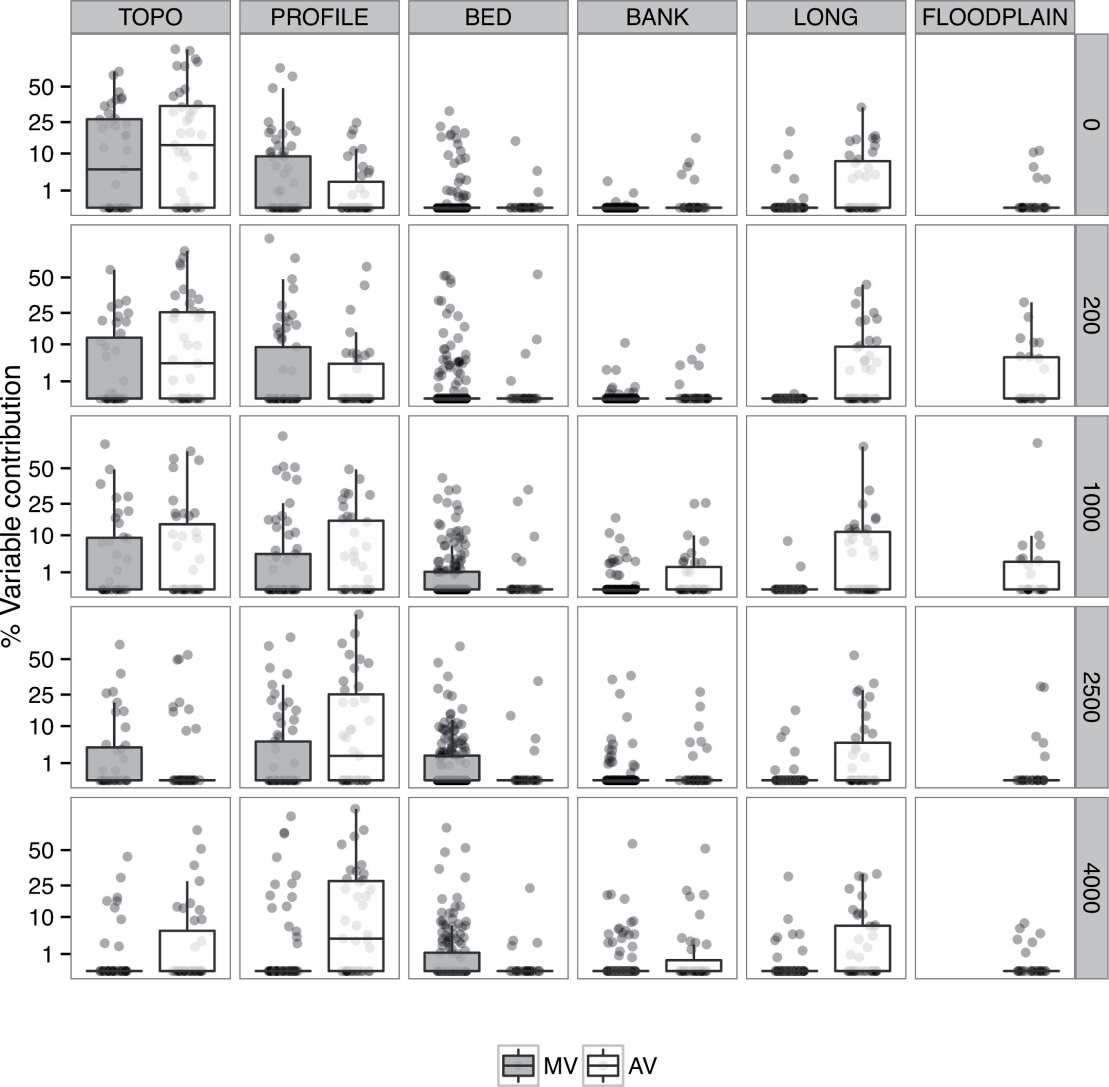
Relative variable contribution to all boosted regression tree models. Relative variable contribution (%) for models including topological, measured (MV, grey boxes) and assessed (AV, white boxes) variables. Results are pooled in five main variable groups (see Table 2) and plotted across five modelled distance classes (0–4000 m). Detailed species-specific information about contributions of the single variables is provided in S1 and S2 Tables (Supporting Information).

The following analysis of variable contribution is based exclusively on models including topological information (n measured = 64, n assessed = 64). Overall, significant differences in the importance of the six groups of variables investigated were detected (Kruskal-Wallis test, χ^2^ = 243.98, df = 5, p-value<0.001) over all modelled distance classes (Fig 3). Topological (TOPO) as well as variables describing the cross-section profile (PROFILE) were best suited to predict the presence of the model fish species. Topological variables (n = 3) were selected 80 times in models based on measured variables and 86 times in models based on assessed variables. This corresponds to a standardized selection frequency (SSF) of 0.41 and 0.44 times that at least one out of all three topological variables has been selected in a single model based on measured and assessed variables respectively. Average per cent variable contribution of TOPO was highest (mean = 10.48, SD = 18.03) and significantly different from the variable groups BANK, BED, FLOODPLAIN and LONG (pairwise Wilcoxon rank sum test, p<0.001) but not from PROFILE (pairwise Wilcoxon rank sum test, p = 0.21). A correlation analysis revealed a strong but non-significant trend of decreasing variable contribution of TOPO with increasing distance classes (Spearman rank correlation, t_3_ = -2.64, p = 0.08, r = -0.84). PROFILE variables (n measured = 5, n assessed = 3) were selected 99 times in models based on measured variables (SSF = 0.30) and 86 times in all models based on assessed variables (SSF = 0.44). Average per cent variable contribution (mean = 7.91, SD = 16.31) significantly differed from the variable groups BANK, BED and LONG (pairwise Wilcoxon rank sum test, p<0.001). In addition, the per cent contribution of assessed variables describing the cross-section profile (PROFILE AV in Fig 3) (mean = 9.73, SD = 17.72) was significantly higher compared to the measured variables (PROFILE MV) (mean = 6.81, SD = 15.34) (two-sided Wilcoxon rank sum test, W = 27453, p<0.005). The importance of variables describing the cross-section profile decreased with increasing distance classes for models based on measured variables (Spearman rank correlation, t_3_ = -1.03, p = 0.37, r = -0.51) whereas it increased for models based on assessed variables (t_3_ = 4.99, p = 0.02, r = 0.94).

LONG variables (n measured = 7, n assessed = 3) were mainly important for models based on assessment scores (85 times selected, SSF = 0.44) but not for models based on measured variables (23 times selected, SSF = 0.05). The per cent contribution of measured LONG variable (mean = 0.31, SD = 2.11) was significantly different from assessed LONG variables (mean = 6.09, SD = 10.75) (two-sided Wilcoxon rank sum test, W = 26777, p<0.001).

BED variables (n measured = 13, n assessed = 2) were selected 208 times in all models based on measured variables (SSF = 0.25) and 20 times in all models based on assessed variables (SSF = 0.15). The per cent variable contribution of BED (mean = 2.43, SD = 7.52) was significantly different from PROFILE and TOPO (pairwise Wilcoxon rank sum test, p<0.001). Only weakly significant differences in the per cent contribution of measured BED (mean = 2.50, SD = 7.58) and assessed BED (mean = 1.98, SD = 7.15) variables could be detected (two-sided Wilcoxon rank sum test, W = 59733, p = 0.03). No significant correlation between the contribution of BED variables and distance classes could be detected for models based on measured and assessed variables (Spearman rank correlation, t_3_ = 1.13, p = 0.34, r = 0.54 resp. t_3_ = -0.40, p = 0.71, r = -0.23).

Floodplain variables (n = 2) where only considered for the models based on assessment scores and were selected only 34 times (SSF = 0.26). Their average per cent variable contribution was generally low (mean = 2.68, SD = 8.30) with slightly higher importance in the distance classes of 200 m and 1000 m.

BANK variables (n measured = 10, n assessed = 3) were selected 66 times (0.10 times / variable x model) and 39 times (0.20 times / variable x model) for models based on measured and assessed variables, respectively. Per cent contribution of measured BANK variable (mean = 0.63, SD = 3.63) significantly differed from assessed BANK variables (mean = 1.81, SD = 5.83) (two-sided Wilcoxon rank sum test, W = 56717, p<0.001)

Extensive and detailed information about the single variables’ contributions and their rank importance for the models using both types of variables is provided in S1 and S2 Tables (Supporting Information).

## Discussion

The main objectives of the study were to test whether the predictive power of species-habitat models for fish increases if (i) habitat conditions at larger spatial scales (up- and downstream of the sampling sites) are considered and (ii) measured hydromorphological variables are used compared to assessed variables, as well as (iii) to identify hydromorphological variables which are best suited to predict the presence of fish species.

### Spatial scales determining the presence of fish

For developing species-habitat models to predict the presence of species, the spatial resolution of the predictors should match the resolution of species’ samplings [41]. The predictors which are predominantly used to develop such models like climate, geology, and elevation [1] vary over large geographical extents and data are usually available at relatively low spatial resolution. In contrast, the hydromorphological data used here were mapped for river reaches down to 100 m, and describe small-scale habitat conditions. This spatial resolution of data might be too high since fish as highly mobile organisms frequently access a wider range of habitats [5,42]. Therefore, the use of small model grid sizes for species-habitat modelling might not adequately account for fishes’ distinct mobility. In a species distribution modelling framework for mobile mammals (bats, *Microchiroptera*), Bellamy et al. [43] have already shown that using so-called focal predictors, which summarize information on environmental variables at larger spatial scales, solves the problem of high-resolution habitat data and describes habitat conditions at the most relevant spatial scale. Similarly, we used focal predictors to account for the distinct mobility of fish and to identify the spatial scale at which hydromorphological conditions determine the presence of fish.

Species dispersal but also spatial correlations of habitats (i.e. environmental gradients caused by common physical forcing) are two main reasons for spatial autocorrelation of species assemblages [44,45]. This study does not explicitly investigate spatial autocorrelation per se (e.g. evaluated by Moran’s I); however, the applied approach might be considered as an indirect way to account for spatial correlations among river fish assemblages by including information about habitat features up- and downstream a site. The use of focal predictors allows to incorporate the species-specific, potentially large movement abilities of riverine fishes [5] in a species-habitat model and thus to account for their ability to utilise multiple, potentially spatially correlated habitats of proximal locations.

As hypothesized, the performance of the models increased by including information on the hydromorphological state up- and downstream a sampling site, similar to other studies which found that fish metrics can be better explained by considering different spatial scales. For example, Wuellner et al. [46] reported improved model performance to predict the presence of prairie fish species if information on reach and catchment scale are combined. Kail and Wolter [8] found significant effects of the hydromorphological state at the site, up- / downstream scale and catchment land use on a set of fish metrics including richness and abundance. Moreover, Ruiz and Peterson [47] hypothesized that (i) the effect of scale depends on the strength of a species’ relationship to local habitat features based on life history requirements and (ii) specialist species are more accurately modelled using local habitat characteristics while generalists are better predicted at larger scales. In our study, model performance improved most by including information on the habitat conditions 2500 m up- and downstream of the sampling sites, indicating that the presence of the fish species modelled depends on the habitats within a river section of 5000 m total length. This result can be used as first estimate for the relevant spatial scale of restoration projects aiming to improve habitat conditions for fish.

However, the presence of fish also depends on other factors besides the hydromorphological state and catchment characteristics, which are typically not considered in statistical models. In our study mean habitat conditions up- and downstream of the sampling sites were calculated. Still, species-habitat associations are even more complex than assuming simple spatial averages based on model raster grid cells. Indeed, species-specific occurrences might also be strongly determined by threshold values of environmental conditions (lower and upper limits of e.g. flow velocity). Moreover, a conversion from vector to raster data is commonly a spatial generalization; however, for this analysis, we don’t assume any related loss of information since the resolution of our raster grid (50 m) is rather fine in comparison the typical lengths of mapped river sections (100 m, [3]).

In addition to the investigated in-stream habitat characteristics, distribution patterns of riverine fish might also be affected by larger-scale pressures such as land use [48] or climate change [49,50] as well as by biotic interactions or invasive species, which are not considered here. Moreover, complex interactions of multiple and interlinked variables and their effects on multiple spatial scales is highly species and life-stage dependent and requires a detailed geo-statistical analysis framework which was beyond the scopes of this study.

### Assessed vs. measured variables

In contrast to our initial expectations, the quantitatively measured in-stream variables did not perform better in predicting species occurrence compared to the general assessment scores. This finding also contradicts the main conclusion of a recent review of more than 50 methods to characterise river habitats by Fernández et al. [9], supposing that quantitatively gathered information could be more effective than evaluation methods as they provide more extensive datasets that could be used for several purposes (e.g. ecological modelling). However, single habitat features are highly variable over time [51] and thus, measurements of single features (e.g. number of riffles) provide just ephemeral snapshots whereas evaluation scores commonly assess the overall state of river compartments like cross-section form or planform (Table 2) which are more constant over time. This might result in less variable model outcomes especially if the fish sampling was not necessarily conducted at the same time as the river habitat survey.

### Habitat variables best suited to predict the presence of fish species

Overall, the hydromorphological data with a high spatial resolution down to 100 m were generally suitable for modelling the presence of riverine fish and depict their typically discontinuous distribution [52]. Almost half of the models (40.8%, n = 106) can be considered “good” (AUC ≥ 0.8) and several (12.7%, n = 33) even “excellent” (AUC≥ 0.9), while only one model failed (AUC < 0.5) [31]. Besides the availability of high resolution hydromorphological data, the good model performance was probably also related to the method used: recent comparisons of methods to model fish species distributions revealed that non-linear approaches (e.g. tree based models such as the applied Boosted Regression Trees) are superior in capturing complex and non-linear patterns in ecological data [53,54]. For our models we used a threshold of one specimen to consider a species present at a sampling site. This accounts for the temporal variation in habitat use and that samplings might miss those periods of high abundance of a species. Selecting a larger presence-threshold (i.e. only sites with higher abundance of a species) and excluding single detections might have resulted in stronger species-habitat associations and better cross-validated model AUCs. In this regard, models considering (relative) species abundances instead of generalized presence/absence data might be valuable approaches to get further insights how species densities might be affected by single habitat features at different spatial scales [55]. However, setting up such abundance-based models typically requires a very specific sampling and analysis design to account for spatial but also temporal patterns in species distributions, which was beyond the scope of this study.

In our study, topological variables describing the river network (stream order) and measured cross section parameters (width, depth, velocity) were the best predictors for the presence of riverine fishes. The high importance of topological variables is in accordance with previous studies showing the high explanatory value of stream order in fish species distribution modelling [31], and the higher importance of the longitudinal gradient over climatic variables for downstream species [56]. Accordingly, we found that stream order variables were especially important for the two ubiquitous species *P*. *fluviatilis* and *R*. *rutilus*, for those most other predictor variables failed (see Supporting Information). Including topological information (e.g. stream order) generally improved the models; however, its relative importance decreased with increasing distance classes while cross section characteristics and streambed features became more important. This supports existing theory that stream order at a specific location within the river network provides already a spatially integrative proxy for the upstream-downstream gradient informing about physical conditions at larger scales [57] and thus gets less important when the same information can be retrieved directly from physical characteristics at this larger scales. The high importance of measured cross section parameters is consistent with findings of Brunke [58] that mean width and flow velocity were the two most important hydromorphological variables that determine fish assemblages in sand dominated rivers in geographical proximity to our study region. In contrast, in heavily degraded river reaches the fish metrics were most strongly related to assessment scores of the riverbank while cross-section parameters were of minor importance [8]. The latter might be simply due to the high uniformity and low variability of cross-sections in heavily degraded reaches (i.e. missing gradient in the dataset).

Besides cross-section parameters, channel-bed characteristics (substrate types) and longitudinal channel features (numbers of river narrowing and islands, river planform) were considered important predictors of fish occurrence. However, this holds true only for the measured channel-bed characteristics and the assessed longitudinal channel features, while both counterparts, assessed channel-bed characteristics and measured longitudinal features, were of minor importance.

This study substantiated the utility of hydromorphological predictor variables and their spatial scaling to improve species-habitat models in lowland sand-dominated streams as shown for the River Treene as study river example. Thus these relationships might also be valid for similar other lowland rivers with comparable sets of fish species and physical habitat characteristics. However, the main findings should be of general applicability that both focal predictors at larger spatial scales and assessment scores perform very well to predict fish species occurrence. Considering the different geomorphological processes [57] and species assemblages [59,60] in other river types (e.g. gravel bed rivers) will probably yield other best suited distance classes for different river types. The further development and application of the proposed focal predictors approach in rivers of different eco-regions would allow to inter-calibrate and to compare species-habitat associations and their spatial aspects between different river types.

Consequently, these two types of habitat characterisations can also be viewed as direct and indirect predictors [61,62] where the measured habitat variables have direct physiological importance to a species and the assessment scores are potentially suited to indirectly evaluate the species-environment relationship [63]. These indirect predictors are potential candidate variables for recent attempts to develop rapid hydromorphological assessment methods, e.g. based on aerial and remote sensing technologies like LiDAR data. This would allow using aerial photography and satellite images in combination with assessment scores to rapidly produce species distribution maps.

### Conclusions

In summary, this study stresses the need to consider habitat conditions up- and downstream of sampling sites in studies investigating species-habitat relationship and thereby acknowledge the distinct mobility of fish. A spatially inclusive and comprehensive dataset on river hydromorphology is a prerequisite to use hydromorphology as a predictor of species occurrence. Such data are increasingly available, especially in European countries, since they are needed for the implementation of the Water Framework Directive. The results indicated that both, measured and assessed variables are suited to predict the presence of fish species and using them in combination is most promising. Moreover, including stream topological variables (e.g. stream order) can substantially improve statistical species-habitat model as they provide valuable additional information describing the relative position of a sampling site within the river network (e.g. temperature gradient, slope, hydrology, hydraulics) which is typically not covered by hydromorphological data. Future studies should focus on identifying indirect hydromorphological predictors which can be measured using remote sensing techniques, and hence, serve as proxies for the hydromorphological conditions in case spatially inclusive and comprehensive ground data are not available.

## Supporting Information

S1 Fig Overview on the overall hydromorphological status of the modelled River Treene and existing migration barriers. (PDF)

S1 Table Relative contribution of each predictor across five focal distance classes using measured hydromorphological variables and with/without topological variables. (PDF)

S2 Table Relative contribution of each predictor across five focal distance classes using assessed hydromorphological variables and with/without topological variables. (PDF)
