# Supplementary material for: Spatial Scaling of Environmental Variables Improves Species-Habitat Models of Fishes in a Small, Sand-Bed Lowland River
Source: PLoS One. 2015 Nov 16;10(11):e0142813. doi: 10.1371/journal.pone.0142813 (PMC4646645; doi:10.1371/journal.pone.0142813)

S1 Figure. Overview on the overall hydromorphological status of the modelled River Treene (catchment: 760 km^2) and existing migration barriers. Large parts of the river have been straightened; 66% of its length is in a poor (orange) or bad (red) hydromorphological state, only some few short near-natural meandering reaches are still present (1.5% of the river length, green). The river network is fragmented by a total of 52 barriers (0.16/km, black triangles). Hydromorphological data were kindly provided by the State Agency for Agriculture, Environment and Rural Areas (LLUR) of the federal state of Schleswig Holstein (status May 2011).

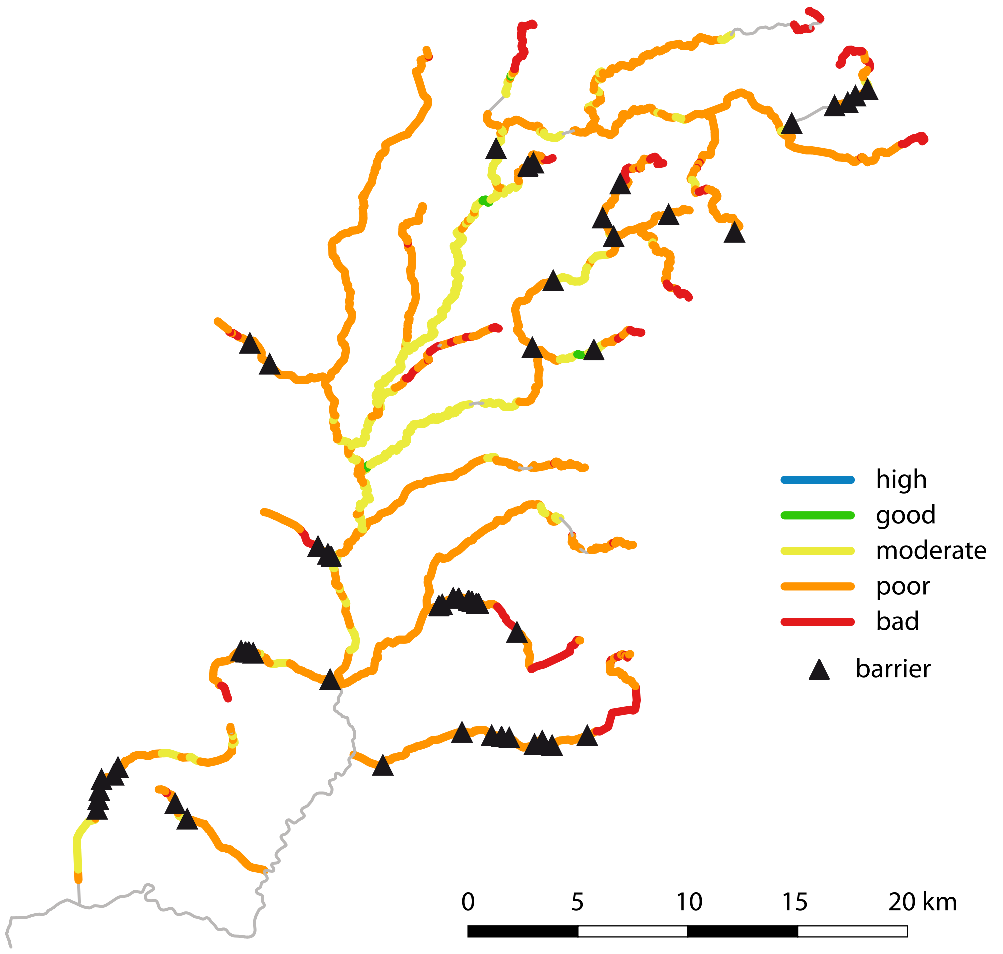

Supplement: S1 Fig — (PDF) [file pone.0142813.s001.pdf]
