# Supplementary material for: Spatial Scaling of Environmental Variables Improves Species-Habitat Models of Fishes in a Small, Sand-Bed Lowland River
Source: PLoS One. 2015 Nov 16;10(11):e0142813. doi: 10.1371/journal.pone.0142813 (PMC4646645; doi:10.1371/journal.pone.0142813)
Supplement: S1 Table — (PDF) [file pone.0142813.s002.pdf]

S1 Table. Relative contribution of each predictor across five focal distance classes using measured hydromorphological variables (MV) and with/without topological variables (TV). Descriptive information about predictor variables and corresponding values are provided in Table 2. Relative contribution (%) measured as the number of times a variable is used for splitting, weighted by the squared improvement at each split and averaged over all trees (Elith et al., 2008). Values in parenthesis indicate importance rank of each variable.

		incl. TV					excl. TV				
Dataset	Variable	0	200	1000	2500	4000	0	200	1000	2500	4000
Anguilla											
TV	DisM	23.73 (1)	7.78 (7)	4.15 (9)	4.30 (8)	0					
TV	SOSh	0	0.13 (19)	0	0	0					
TV	SOSt	18.21 (3)	9.12 (4)	7.46 (6)	0.31 (20)	0					
MV	BAEr	0	0.16 (18)	1.12 (14)	2.23 (14)	0	0	0	0	2.02 (13)	0
MV	BAOt	0	2.14 (13)	0	0	0	0	0	0	1.58 (14)	0
MV	BAWa	0	0.40 (16)	0	1.00 (17)	0	0	0	0	0.99 (16)	0
MV	BFLW	0	0.07 (22)	0	0	0	0	0	0	0	0
MV	BFOt	0	0	0	1.23 (15)	0	0	0	0	0.94 (17)	0
MV	BPGr	0	0	0	0	0	0	0	0	0	0
MV	BPMa	0	0	0	0	0	0	0	0	0	0
MV	BPno	0	0.30 (17)	2.92 (10)	0.92 (18)	0	0	0	6.04 (8)	0.88 (18)	0
MV	BPRi	0	0	0	0	0	0	0	0	0	0
MV	BPWo	0	0.09 (20)	2.68 (11)	3.09 (9)	17.72 (5)	0	0	0	2.84 (10)	0
MV	CBFO	0	0	0	0	0	0	0	0	0	0
MV	CBFR	0	1.13 (15)	0	2.62 (12)	0	0	0	0	3.20 (8)	0
MV	CFIB	0	0	0	0.35 (19)	0	0	0	0	0	0
MV	CFLW	0	0.00 (23)	0	0	0	0	0	0	0	0
MV	CFNa	0	0	0	0	0	0	0	0	0	0
MV	CFWi	0	0.07 (21)	0	0	0	0	0	0	0	0
MV	ChDe	19.24 (2)	9.03 (5)	4.28 (8)	20.79 (1)	26.16 (1)	27.03 (2)	0	6.66 (6)	22.12 (1)	64.17 (1)
MV	ChDV	0	0	0	2.79 (11)	0	0	0	0	2.82 (11)	0
MV	ChWi	13.14 (5)	17.00 (1)	16.53 (2)	16.64 (2)	18.70 (3)	36.80 (1)	60.12 (1)	19.16 (1)	17.80 (2)	35.83 (2)
MV	ChWV	0	0	0	0	0	0	0	0	0	0
MV	CSFo	0	0	0	0	0	0	0	0	0	0
MV	FlDi	0	0	0	0	0	0	0	0	0	0
MV	FlVe	0	0	0	0	0	0	0	0	0	0
MV	InVe	0	0	0	0	0	0	0	0	0	0
MV	Plan	0	0	0	0	0	0	0	0	0	0
MV	RVRe	0	0	0	0	0	0	0	0	0	0
MV	RVSp	0	2.83 (12)	5.04 (7)	1.12 (16)	0	0	0	6.63 (7)	1.03 (15)	0
MV	RVTF	0	10.55 (3)	17.47 (1)	5.80 (6)	19.70 (2)	0	0	16.67 (2)	5.44 (6)	0
MV	SMaS	0	4.52 (10)	11.92 (4)	2.79 (10)	0	0	0	14.03 (4)	2.43 (12)	0

		incl. TV					excl. TV				
Dataset	Variable	0	200	1000	2500	4000	0	200	1000	2500	4000
MV	SuDi	17.80 (4)	12.42 (2)	7.63 (5)	5.37 (7)	0	22.76 (3)	39.88 (2)	10.54 (5)	6.11 (5)	0
MV	SuHa	0	1.83 (14)	0	6.03 (5)	0	0	0	0	5.43 (7)	0
MV	SuMa	0	4.74 (8)	14.69 (3)	8.58 (4)	17.72 (4)	0	0	16.15 (3)	9.15 (4)	0
MV	SuSa	7.89 (6)	4.57 (9)	1.07 (15)	0	0	13.40 (4)	0	0	0	0
MV	SuSo	0	2.89 (11)	1.24 (13)	2.56 (13)	0	0	0	2.39 (9)	2.93 (9)	0
MV	SuWo	0	8.26 (6)	1.80 (12)	11.47 (3)	0	0	0	1.72 (10)	12.30 (3)	0
Cobienia											
TV	DisM	21.44 (2)	0	15.00 (3)	0	2.09 (18)					
TV	SOSh	22.95 (1)	30.59 (2)	19.83 (1)	0	0					
TV	SOSt	12.35 (5)	0	9.31 (7)	38.78 (2)	13.70 (1)					
MV	BAEr	0	0	0	0	3.56 (13)	0	0	0	3.97 (9)	0
MV	BAOt	0	0	17.71 (2)	0	5.11 (7)	0	0	0	5.66 (6)	8.50 (7)
MV	BAWa	0	0	0	0	0.61 (24)	0	0	0	0	0
MV	BFLW	0	0	0	0	0.83 (22)	0	0	0	18.73 (1)	0
MV	BFOt	0	0	0	0	0	0	0	0	0	0
MV	BPGr	0	0	0	0	0	0	0	0	0	0
MV	BPMa	0	0	0	0	0	0	0	0	0	0
MV	BPno	0	0	0	0	4.51 (9)	0	0	0	15.08 (2)	10.33 (6)
MV	BPRi	0	0	0	0	6.24 (6)	0	0	0	0	0
MV	BPWo	0	0	0	0	0	0	0	0	0	0
MV	CBFO	0	0	0	0	7.21 (4)	0	0	0	0	11.95 (4)
MV	CBFR	0	0	0	0	1.15 (20)	0	0	0	0	0
MV	CFIB	0	0	0	0	9.23 (2)	0	0	0	3.22 (12)	16.95 (1)
MV	CFLW	0	0	0	0	3.23 (14)	0	0	0	0	0
MV	CFNa	0	0	0	0	3.88 (12)	0	0	0	0	0
MV	CFWi	0	0	0	0	1.43 (19)	0	0	0	0	0
MV	ChDe	0	0	0	0	4.00 (10)	0	0	0	4.63 (8)	13.81 (3)
MV	ChDV	0	0	0	0	0	0	0	0	6.10 (5)	0
MV	ChWi	15.87 (4)	23.74 (3)	13.23 (5)	0	2.60 (17)	55.18 (1)	48.73 (2)	56.27 (1)	14.00 (4)	8.00 (8)
MV	ChWV	0	0	0	0	0	0	0	0	0	0
MV	CSFo	0	0	0	0	0	0	0	0	0	0
MV	FlDi	0	0	0	0	0	0	0	0	0	0
MV	FlVe	0	0	0	0	0	0	0	0	0	0
MV	InVe	0	0	0	0	0	0	0	0	0	0
MV	Plan	0	0	0	0	0	0	0	0	0	0
MV	RVRe	0	0	0	0	0	0	0	0	0	0
MV	RVSp	0	0	0	0	0.64 (23)	0	0	0	0	0
MV	RVTF	0	0	0	0	2.85 (15)	0	0	0	1.58 (13)	5.02 (9)

		incl. TV					excl. TV				
Dataset	Variable	0	200	1000	2500	4000	0	200	1000	2500	4000
MV	SMaS	0	0	0	0	0	0	0	0	0	0
MV	SuDi	18.96 (3)	45.67 (1)	14.02 (4)	0	2.83 (16)	35.53 (2)	51.27 (1)	43.73 (2)	3.89 (10)	0
MV	SuHa	8.43 (6)	0	0	0	4.73 (8)	9.29 (3)	0	0	0	0
MV	SuMa	0	0	0	0	0.98 (21)	0	0	0	0	0
MV	SuSa	0	0	10.90 (6)	0	3.90 (11)	0	0	0	3.83 (11)	0
MV	SuSo	0	0	0	61.22 (1)	6.36 (5)	0	0	0	14.52 (3)	11.60 (5)
MV	SuWo	0	0	0	0	8.33 (3)	0	0	0	4.79 (7)	13.84 (2)
Gastatus											
TV	DisM	41.27 (2)	0	7.79 (4)	3.24 (6)	0					
TV	SOSh	0	0	0	0	0					
TV	SOSt	0	0	3.12 (9)	0.30 (22)	0					
MV	BAEr	0	0	2.32 (12)	1.91 (12)	0	0	0	0	0	0
MV	BAOt	0	0	0	2.06 (11)	0	0	0	0	0	0
MV	BAWa	0	0	2.62 (10)	0.74 (18)	0	0	0	0	0	0
MV	BFLW	0	0	0	0.67 (19)	0	0	0	0	0	0
MV	BFOt	0	0	0	0	0	0	0	0	0	0
MV	BPGr	0	0	0	0	0	0	0	0	0	0
MV	BPMa	0	0	0	0	0	0	0	0	0	0
MV	BPno	0	0	10.85 (2)	0.83 (16)	0	0	0	19.26 (2)	0	0
MV	BPRi	0	0	0	0	0	0	0	0	0	0
MV	BPWo	0	0	0	0	0	0	0	0	0	0
MV	CBFO	0	0	0	0	0	0	0	0	0	0
MV	CBFR	0	0	0.76 (16)	2.83 (7)	18.35 (2)	0	0	0	0	22.35 (2)
MV	CFIB	0	0	0	0	0	0	0	0	0	0
MV	CFLW	0	0	0	0	0	0	0	0	0	0
MV	CFNa	0	0	0	0	0	0	0	0	0	0
MV	CFWi	0	0	0	16.72 (2)	0	0	0	0	0	0
MV	ChDe	0	0	3.76 (7)	6.35 (3)	0	0	0	14.30 (4)	26.49 (2)	0
MV	ChDV	0	0	0	0.42 (21)	0	0	0	0	0	0
MV	ChWi	58.73 (1)	67.26 (1)	40.98 (1)	43.06 (1)	81.65 (1)	85.01 (1)	67.16 (1)	51.69 (1)	73.51 (1)	77.65 (1)
MV	ChWV	0	0	0	0.98 (14)	0	0	0	0	0	0
MV	CSFo	0	0	0	0.57 (20)	0	14.99 (2)	0	0	0	0
MV	FlDi	0	0	0	0	0	0	0	0	0	0
MV	FlVe	0	0	0	0	0	0	0	0	0	0
MV	InVe	0	0	0	0	0	0	0	0	0	0
MV	Plan	0	0	0	0	0	0	0	0	0	0
MV	RVRe	0	0	0	0	0	0	0	0	0	0
MV	RVSp	0	0	2.46 (11)	0.18 (23)	0	0	0	0	0	0

		incl. TV					excl. TV				
Dataset	Variable	0	200	1000	2500	4000	0	200	1000	2500	4000
MV	RVTF	0	0	3.16 (8)	1.67 (13)	0	0	0	0	0	0
MV	SMaS	0	0	0.87 (15)	3.59 (5)	0	0	0	0	0	0
MV	SuDi	0	0	4.49 (6)	2.53 (9)	0	0	0	0	0	0
MV	SuHa	0	32.74 (2)	1.01 (14)	0.93 (15)	0	0	32.84 (2)	0	0	0
MV	SuMa	0	0	1.13 (13)	0.75 (17)	0	0	0	0	0	0
MV	SuSa	0	0	8.57 (3)	2.17 (10)	0	0	0	14.75 (3)	0	0
MV	SuSo	0	0	6.13 (5)	2.75 (8)	0	0	0	0	0	0
MV	SuWo	0	0	0	4.75 (4)	0	0	0	0	0	0
Gobiobio											
TV	DisM	0	0	0	0	0					
TV	SOSh	0	0	0	0	0					
TV	SOSt	5.01 (4)	0	1.23 (6)	1.59 (6)	2.63 (5)					
MV	BAEr	0	0	0	0	0	0	0	0	0	0
MV	BAOt	0	0	0	0	0	0	0	12.23 (2)	0	0
MV	BAWa	0	0	0	0	0	0	0	0	0	0
MV	BFLW	0	0	0	0	0	0	0	0	0	0
MV	BFOt	0	0	0	0	1.33 (7)	0	0	0	0	0
MV	BPGr	0	0	0	0	0	0	0	0	0	0
MV	BPMa	0	0	0	0	0	0	0	0	0	0
MV	BPno	0	0	0	0	0	0	0	0	0	0
MV	BPRi	0	0	0	0	0	0	0	0	0	0
MV	BPWo	0	0	0	0	0	0	0	0	0	0
MV	CBFO	0	0	0	0	0	0	0	0	0	0
MV	CBFR	0	0	0	0	0	0	0	0	0	0
MV	CFIB	0	0	0	0	0	0	0	0	0	0
MV	CFLW	0	0	0	0	0	0	0	0	0	0
MV	CFNa	0	0	0	0	0	0	0	0	0	0
MV	CFWi	0	0	0	0	0	0	0	0	0	0
MV	ChDe	5.92 (3)	12.63 (2)	2.91 (4)	7.04 (3)	14.27 (2)	5.47 (3)	12.38 (2)	1.92 (6)	7.27 (4)	18.95 (2)
MV	ChDV	0	0	0	0	0	0	0	0	0	0
MV	ChWi	66.59 (1)	87.37 (1)	80.14 (1)	69.74 (1)	65.42 (1)	73.58 (1)	87.62 (1)	74.79 (1)	69.29 (1)	81.05 (1)
MV	ChWV	0	0	0	0	0	0	0	0	0	0
MV	CSFo	0	0	0	0	0	0	0	0	0	0
MV	FlDi	0	0	0	0	0	0	0	0	0	0
MV	FlVe	0	0	0	0	0	0	0	0	0	0
MV	InVe	0	0	0	0	0	0	0	0	0	0
MV	Plan	0	0	0	0	0	0	0	0	0	0
MV	RVRe	0	0	0	0	0	0	0	0	0	0

		incl. TV					excl. TV				
Dataset	Variable	0	200	1000	2500	4000	0	200	1000	2500	4000
MV	RVSp	0	0	2.30 (5)	0	0	0	0	2.98 (4)	0	0
MV	RVTF	0	0	0	0	0	0	0	0	0	0
MV	SMaS	0	0	0	0	7.12 (4)	0	0	0	0	0
MV	SuDi	0	0	0	10.68 (2)	0	0	0	0	11.01 (2)	0
MV	SuHa	0	0	0	0	0	0	0	0	0	0
MV	SuMa	0	0	0	0	0	0	0	0	0	0
MV	SuSa	0	0	6.88 (2)	6.25 (4)	7.56 (3)	0	0	5.57 (3)	8.37 (3)	0
MV	SuSo	22.48 (2)	0	6.53 (3)	4.70 (5)	1.67 (6)	20.95 (2)	0	2.52 (5)	0	0
MV	SuWo	0	0	0	0	0	0	0	0	4.06 (5)	0
Gymnrnua											
TV	DisM	35.25 (2)	19.69 (3)	17.36 (2)	15.95 (2)	18.45 (4)					
TV	SOSh	37.07 (1)	20.69 (2)	28.74 (1)	20.62 (1)	0					
TV	SOSt	0	0	0	0	0					
MV	BAEr	0	0	0	8.72 (5)	0	0	0	0	12.28 (5)	0
MV	BAOt	0	0	11.66 (3)	6.99 (7)	0	0	0	0	10.66 (6)	10.57 (5)
MV	BAWa	0	0	0	0	0	0	0	0	0	0
MV	BFLW	0	0	0	13.61 (3)	19.98 (2)	0	0	0	20.34 (1)	17.44 (3)
MV	BFOt	0	0	0	0	0	0	0	0	0	0
MV	BPGr	0	0	0	0	0	0	0	0	0	0
MV	BPMa	0	0	0	0	0	0	0	0	0	0
MV	BPno	0	0	0	0	0	0	0	0	0	0
MV	BPRi	0	0	0	0	0	0	0	0	0	0
MV	BPWo	0	0	0	0	0	0	0	0	0	0
MV	CBFO	0	0	0	0	0	0	0	0	0	0
MV	CBFR	0	0	0	0	0	0	0	0	0	0
MV	CFIB	0	0	0	0	0	0	0	0	0	0
MV	CFLW	0	0	0	0	30.51 (1)	0	0	0	0	23.72 (1)
MV	CFNa	0	0	0	0	0	0	0	0	0	0
MV	CFWi	0	0	0	0	0	0	0	0	0	0
MV	ChDe	8.99 (4)	15.05 (4)	5.19 (8)	8.51 (6)	0	23.80 (2)	29.13 (2)	26.00 (2)	16.67 (3)	4.03 (7)
MV	ChDV	0	0	0	0	0	0	0	0	0	0
MV	ChWi	18.69 (3)	11.86 (5)	10.12 (5)	5.13 (8)	19.48 (3)	76.20 (1)	23.73 (3)	49.04 (1)	15.26 (4)	19.40 (2)
MV	ChWV	0	0	0	0	0	0	0	0	0	0
MV	CSFo	0	0	0	0	0	0	0	0	0	0
MV	FlDi	0	0	0	0	0	0	0	0	0	0
MV	FlVe	0	0	0	0	0	0	0	0	0	0
MV	InVe	0	0	0	0	0	0	0	0	0	0
MV	Plan	0	0	0	0	0	0	0	0	0	0

		incl. TV					excl. TV				
Dataset	Variable	0	200	1000	2500	4000	0	200	1000	2500	4000
MV	RVRe	0	0	0	0	0	0	0	0	0	0
MV	RVSp	0	0	0	0	0	0	0	0	0	0
MV	RVTF	0	0	0	3.31 (10)	0	0	0	0	0	0
MV	SMaS	0	0	11.43 (4)	0	0	0	0	0	0	0
MV	SuDi	0	27.59 (1)	0	12.56 (4)	0	0	38.57 (1)	0	16.86 (2)	16.81 (4)
MV	SuHa	0	0	0	0	0	0	0	0	0	0
MV	SuMa	0	5.12 (6)	8.96 (6)	0	0	0	8.57 (4)	24.96 (3)	0	0
MV	SuSa	0	0	6.55 (7)	0	0	0	0	0	0	0
MV	SuSo	0	0	0	4.60 (9)	11.57 (5)	0	0	0	7.94 (7)	8.04 (6)
MV	SuWo	0	0	0	0	0	0	0	0	0	0
Leucscus											
TV	DisM	27.01 (1)	24.88 (1)	29.48 (2)	0	0					
TV	SOSh	0	0	0	0	0					
TV	SOSt	0	0	0	0	0					
MV	BAEr	0	0	0	0	0	0	0	0	0	0
MV	BAOt	0	0	0	0	0	0	0	0	0	0
MV	BAWa	0	0	0	0	0	0	0	0	0	0
MV	BFLW	0	0	0	0	0	0	0	0	0	0
MV	BFOt	0	0	0	0	0	0	0	0	0	19.01 (3)
MV	BPGr	0	0	0	0	0	0	0	0	0	0
MV	BPMa	0	0	0	0	0	0	0	0	0	0
MV	BPno	0	0	0	0	0	0	0	0	0	0
MV	BPRi	0	0	0	0	0	0	0	0	0	0
MV	BPWo	0	0	0	0	0	0	0	0	0	0
MV	CBFO	0	0	0	0	0	0	0	0	0	0
MV	CBFR	0	0	0	0	0	0	0	0	0	0
MV	CFIB	0	0	0	0	0	0	0	0	0	0
MV	CFLW	0	0	0	0	0	0	0	0	0	0
MV	CFNa	0	0	0	0	0	0	0	0	0	0
MV	CFWi	0	0	0	0	0	0	0	0	0	0
MV	ChDe	8.78 (5)	18.47 (4)	11.04 (4)	18.71 (4)	0	15.35 (4)	22.54 (3)	16.19 (3)	19.31 (4)	0
MV	ChDV	0	0	0	0	0	0	0	0	0	0
MV	ChWi	19.99 (3)	22.50 (3)	25.41 (3)	25.37 (1)	64.42 (1)	27.36 (1)	29.87 (2)	33.34 (2)	24.55 (2)	50.58 (1)
MV	ChWV	0	0	0	0	0	0	0	0	0	0
MV	CSFo	20.86 (2)	0	0	21.79 (3)	0	24.83 (2)	0	0	22.18 (3)	0
MV	FlDi	0	0	0	0	0	0	0	0	0	0
MV	FlVe	0	0	0	0	0	0	0	0	0	0
MV	InVe	0	0	0	0	0	0	0	0	0	0

		incl. TV					excl. TV				
Dataset	Variable	0	200	1000	2500	4000	0	200	1000	2500	4000
MV	Plan	0	0	0	0	0	0	0	0	0	0
MV	RVRe	0	0	0	0	0	0	0	0	0	0
MV	RVSp	0	0	0	0	0	0	0	0	0	0
MV	RVTF	0	0	0	0	0	0	0	0	0	0
MV	SMaS	0	0	0	0	0	0	0	0	0	0
MV	SuDi	0	0	0	0	0	0	0	0	0	0
MV	SuHa	0	0	0	0	0	0	0	0	0	0
MV	SuMa	0	0	0	0	0	0	0	0	0	0
MV	SuSa	6.76 (6)	9.61 (5)	0	9.07 (5)	0	10.00 (5)	16.83 (4)	0	8.94 (5)	0
MV	SuSo	16.60 (4)	24.53 (2)	34.06 (1)	25.07 (2)	35.58 (2)	22.46 (3)	30.76 (1)	50.47 (1)	25.02 (1)	30.41 (2)
MV	SuWo	0	0	0	0	0	0	0	0	0	0
Percilis											
TV	DisM	0	0	0	2.94 (10)	0					
TV	SOSh	39.98 (2)	23.00 (3)	37.86 (2)	4.14 (8)	16.74 (2)					
TV	SOSt	11.24 (3)	6.02 (4)	0	9.56 (3)	9.11 (3)					
MV	BAEr	0	0	0	8.66 (5)	3.58 (8)	0	0	0	0	4.56 (7)
MV	BAOt	0	0	0	2.22 (12)	0	0	0	0	0	0
MV	BAWa	0	0	0	1.31 (14)	1.14 (16)	0	0	0	0	1.56 (10)
MV	BFLW	0	0	0	4.44 (6)	4.62 (6)	0	0	0	0	6.04 (5)
MV	BFOt	0	0	0	0	0	0	0	0	0	0
MV	BPGr	0	0	0	0	0	0	0	0	0	0
MV	BPMa	0	0	0	0	0	0	0	0	0	0
MV	BPno	0	0	0	0	0	0	0	0	0	0
MV	BPRi	0	0	0	0	0	0	0	0	0	0
MV	BPWo	0	0	0	2.01 (13)	0	0	0	0	0	0
MV	CBFO	0	0	0	0	3.85 (7)	0	0	0	0	8.32 (4)
MV	CBFR	0	0	0	0	1.89 (14)	0	0	0	0	0
MV	CFIB	0	0	0	0	0	0	0	0	0	0
MV	CFLW	0	0	0	0	0.64 (17)	0	0	0	0	0
MV	CFNa	0	0	0	0	0	0	0	0	0	0
MV	CFWi	0	0	0	0	0	0	0	0	0	0
MV	ChDe	0	29.70 (2)	18.48 (3)	13.83 (2)	6.78 (4)	8.42 (4)	36.53 (2)	23.22 (2)	31.55 (2)	10.44 (2)
MV	ChDV	0	0	0	0	0	0	0	0	0	0
MV	ChWi	48.78 (1)	41.28 (1)	43.66 (1)	31.09 (1)	31.16 (1)	46.72 (1)	63.47 (1)	76.78 (1)	68.45 (1)	46.20 (1)
MV	ChWV	0	0	0	0	0	0	0	0	0	0
MV	CSFo	0	0	0	0	0	7.90 (6)	0	0	0	0
MV	FlDi	0	0	0	0	0	0	0	0	0	0
MV	FlVe	0	0	0	0	0	0	0	0	0	0

		incl. TV					excl. TV				
Dataset	Variable	0	200	1000	2500	4000	0	200	1000	2500	4000
MV	InVe	0	0	0	0	0	0	0	0	0	0
MV	Plan	0	0	0	0	0	8.16 (5)	0	0	0	0
MV	RVRe	0	0	0	0	0	0	0	0	0	0
MV	RVSp	0	0	0	0	2.73 (10)	0	0	0	0	0
MV	RVTF	0	0	0	0	2.05 (13)	0	0	0	0	0
MV	SMaS	0	0	0	3.90 (9)	2.62 (11)	0	0	0	0	5.39 (6)
MV	SuDi	0	0	0	8.82 (4)	6.56 (5)	10.23 (3)	0	0	0	9.92 (3)
MV	SuHa	0	0	0	0	0	0	0	0	0	0
MV	SuMa	0	0	0	0	0	0	0	0	0	0
MV	SuSa	0	0	0	2.68 (11)	1.42 (15)	5.37 (7)	0	0	0	3.16 (9)
MV	SuSo	0	0	0	4.42 (7)	3.00 (9)	13.20 (2)	0	0	0	4.42 (8)
MV	SuWo	0	0	0	0	2.11 (12)	0	0	0	0	0
Phoxinus											
TV	DisM	26.42 (1)	20.17 (2)	0	0	0					
TV	SOSh	17.56 (2)	10.93 (4)	0	0	0					
TV	SOSt	1.80 (11)	1.06 (14)	0	0	0					
MV	BAEr	0.05 (25)	0	0	0	0	0.06 (23)	0	0	0	0
MV	BAOt	0.00 (28)	0	0	0	0	0.01 (24)	0	0	0	0
MV	BAWa	0.32 (21)	2.46 (11)	0	0	0	0.55 (18)	0	0	0	0
MV	BFLW	0.00 (32)	0	0	0	0	0	0	0	0	0
MV	BFOt	0.00 (33)	0	0	0	0	0	0	0	0	0
MV	BPGr	0	0	0	0	0	0	0	0	0	0
MV	BPMa	0	0	0	0	0	0	0	0	0	0
MV	BPno	0.11 (23)	0.42 (15)	0	0	0	0.28 (19)	0	0	0	0
MV	BPRi	0	0	0	0	0	0	0	0	0	0
MV	BPWo	2.40 (9)	2.77 (10)	0	0	0	5.48 (5)	0	0	0	0
MV	CBFO	0	0	0	0	0	0	0	0	0	0
MV	CBFR	0.40 (20)	0.20 (16)	0	0	0	0.58 (17)	0	0	0	0
MV	CFIB	0.00 (30)	0	0	0	0	0.00 (27)	0	0	0	0
MV	CFLW	0.00 (29)	0	0	0	0	0.00 (26)	0	0	0	0
MV	CFNa	0.07 (24)	0	0	0	0	0.17 (20)	0	0	0	0
MV	CFWi	0.00 (31)	0	0	0	0	0.00 (28)	0	0	0	0
MV	ChDe	10.77 (3)	13.84 (3)	48.56 (2)	38.52 (2)	25.67 (2)	15.00 (3)	38.27 (2)	40.07 (1)	40.44 (2)	25.02 (2)
MV	ChDV	0.30 (22)	0	0	0	0	0.15 (21)	0	0	0	0
MV	ChWi	9.31 (5)	22.86 (1)	51.44 (1)	61.48 (1)	44.16 (1)	29.89 (1)	61.73 (1)	37.81 (2)	59.56 (1)	43.57 (1)
MV	ChWV	0.97 (13)	0	0	0	0	1.54 (13)	0	0	0	0
MV	CSFo	0.92 (15)	0	0	0	0	1.51 (14)	0	0	0	0
MV	FlDi	0.00 (27)	0	0	0	0	0.00 (25)	0	0	0	0

		incl. TV					excl. TV				
Dataset	Variable	0	200	1000	2500	4000	0	200	1000	2500	4000
MV	FlVe	10.74 (4)	0	0	0	0	16.17 (2)	0	0	0	0
MV	InVe	2.23 (10)	0	0	0	0	2.89 (7)	0	0	0	0
MV	Plan	5.18 (6)	0	0	0	0	9.10 (4)	0	0	0	0
MV	RVRe	0	0	0	0	0	0	0	0	0	0
MV	RVSp	0.75 (17)	0	0	0	0	2.17 (8)	0	0	0	0
MV	RVTF	0.02 (26)	0	0	0	0	0.11 (22)	0	0	0	0
MV	SMaS	0.51 (19)	3.78 (9)	0	0	0	1.72 (12)	0	0	0	0
MV	SuDi	2.62 (8)	3.79 (8)	0	0	4.68 (5)	5.02 (6)	0	0	0	4.65 (5)
MV	SuHa	0.85 (16)	2.35 (12)	0	0	0	1.20 (15)	0	0	0	0
MV	SuMa	0.59 (18)	4.59 (6)	0	0	0	0.78 (16)	0	0	0	0
MV	SuSa	0.95 (14)	1.64 (13)	0	0	11.42 (4)	2.09 (9)	0	22.11 (3)	0	11.70 (4)
MV	SuSo	1.05 (12)	4.17 (7)	0	0	0	1.78 (10)	0	0	0	0
MV	SuWo	3.12 (7)	4.97 (5)	0	0	14.07 (3)	1.75 (11)	0	0	0	15.06 (3)
Pungtius											
TV	DisM	30.41 (1)	0	0	0	0					
TV	SOSh	0	0	0	0	0					
TV	SOSt	0	0	0	0	0					
MV	BAEr	0	0	0	0	29.96 (2)	0	0	0	0	27.81 (2)
MV	BAOt	0	0	0	0	0	0	0	0	0	0
MV	BAWa	0	0	0	0	0	0	0	0	0	0
MV	BFLW	0	0	0	0	0	0	0	0	0	0
MV	BFOt	0	0	0	0	0	0	0	0	0	0
MV	BPGr	0	0	0	0	0	0	0	0	0	0
MV	BPMa	0	0	0	0	0	0	0	0	0	0
MV	BPno	0	0	0	0	0	0	0	0	0	0
MV	BPRi	0	0	0	0	0	0	0	0	0	0
MV	BPWo	0	0	0	0	0	0	0	0	0	0
MV	CBFO	0	0	0	0	0	0	0	0	0	0
MV	CBFR	0	0	24.38 (3)	24.21 (3)	0	0	0	19.60 (2)	30.01 (2)	0
MV	CFIB	0	0	0	0	0	0	0	0	0	0
MV	CFLW	0	0	0	0	0	0	0	0	0	0
MV	CFNa	0	0	0	0	0	0	0	0	0	0
MV	CFWi	0	0	0	0	0	0	0	0	0	0
MV	ChDe	0	0	0	0	0	0	0	10.02 (5)	0	0
MV	ChDV	0	0	0	0	0	0	0	0	0	0
MV	ChWi	12.98 (5)	48.65 (2)	16.56 (4)	0	0	21.15 (4)	31.01 (1)	9.43 (6)	0	0
MV	ChWV	0	0	0	0	0	0	0	0	0	0
MV	CSFo	22.95 (2)	0	0	0	0	28.49 (1)	0	0	0	0

		incl. TV					excl. TV				
Dataset	Variable	0	200	1000	2500	4000	0	200	1000	2500	4000
MV	FlDi	0	0	0	0	0	0	0	0	0	0
MV	FlVe	0	0	0	0	0	0	0	0	0	0
MV	InVe	0	0	0	0	0	0	0	0	0	0
MV	Plan	19.81 (3)	0	0	0	0	26.18 (2)	0	0	0	0
MV	RVRe	0	0	0	0	0	0	0	0	0	0
MV	RVSp	0	0	0	0	0	0	0	0	0	0
MV	RVTF	0	0	0	0	0	0	0	0	0	0
MV	SMaS	0	0	0	0	0	0	0	0	0	0
MV	SuDi	0	0	0	0	0	0	20.27 (3)	0	0	0
MV	SuHa	0	0	0	0	0	0	0	0	0	0
MV	SuMa	0	0	0	0	0	0	19.69 (4)	13.76 (4)	0	0
MV	SuSa	13.86 (4)	51.35 (1)	25.66 (2)	47.17 (1)	70.04 (1)	24.18 (3)	29.03 (2)	18.73 (3)	69.99 (1)	72.19 (1)
MV	SuSo	0	0	0	0	0	0	0	0	0	0
MV	SuWo	0	0	33.41 (1)	28.61 (2)	0	0	0	28.46 (1)	0	0
Rutiilus											
TV	DisM	0	0	0	0	0					
TV	SOSh	40.12 (2)	14.42 (4)	0	0	0					
TV	SOSt	59.88 (1)	32.65 (1)	49.12 (2)	26.70 (2)	29.76 (1)					
MV	BAEr	0	0	0	0	0	0	0	0	0	0
MV	BAOt	0	0	0	0	0	0	0	0	0	0
MV	BAWa	0	0	0	0	8.42 (5)	0	0	0	0	0
MV	BFLW	0	0	0	0	4.67 (9)	0	0	0	0	0
MV	BFOt	0	0	0	0	0	0	0	0	0	0
MV	BPGr	0	0	0	0	0	0	0	0	0	0
MV	BPMa	0	0	0	0	0	0	0	0	0	0
MV	BPno	0	0	0	25.46 (3)	8.51 (4)	0	0	0	28.32 (3)	0
MV	BPRi	0	0	0	0	0	0	0	0	0	0
MV	BPWo	0	0	0	0	4.98 (8)	0	0	0	0	0
MV	CBFO	0	0	0	0	14.41 (2)	0	0	0	0	0
MV	CBFR	0	0	0	0	0	0	0	0	0	0
MV	CFIB	0	0	0	0	0	0	0	0	0	0
MV	CFLW	0	0	0	0	0	0	0	0	0	0
MV	CFNa	0	0	0	0	4.66 (10)	0	0	0	0	0
MV	CFWi	0	0	0	0	0	0	0	0	0	0
MV	ChDe	0	0	0	0	0	0	0	0	0	0
MV	ChDV	0	0	0	0	0	0	0	0	0	0
MV	ChWi	0	20.48 (3)	50.88 (1)	11.09 (4)	5.38 (7)	50.15 (1)	62.21 (1)	57.58 (1)	30.14 (2)	50.83 (1)
MV	ChWV	0	0	0	0	0	0	0	0	0	0

		incl. TV					excl. TV				
Dataset	Variable	0	200	1000	2500	4000	0	200	1000	2500	4000
MV	CSFo	0	0	0	0	0	0	0	0	0	0
MV	FlDi	0	0	0	0	0	0	0	0	0	0
MV	FlVe	0	0	0	0	0	0	0	0	0	0
MV	InVe	0	0	0	0	0	0	0	0	0	0
MV	Plan	0	0	0	0	0	0	0	0	0	0
MV	RVRe	0	0	0	0	0	0	0	0	0	0
MV	RVSp	0	0	0	0	5.56 (6)	0	0	0	0	0
MV	RVTF	0	0	0	0	0	0	0	0	0	0
MV	SMaS	0	0	0	36.75 (1)	9.33 (3)	0	0	42.42 (2)	41.54 (1)	49.17 (2)
MV	SuDi	0	0	0	0	0	29.54 (2)	0	0	0	0
MV	SuHa	0	0	0	0	0	0	0	0	0	0
MV	SuMa	0	0	0	0	0	0	0	0	0	0
MV	SuSa	0	0	0	0	0	0	0	0	0	0
MV	SuSo	0	32.45 (2)	0	0	0	20.31 (3)	37.79 (2)	0	0	0
MV	SuWo	0	0	0	0	4.32 (11)	0	0	0	0	0
Salmalar											
TV	DisM	0	0	0	0	0					
TV	SOSh	63.18 (1)	56.49 (1)	71.99 (1)	62.70 (1)	44.73 (2)					
TV	SOSt	0	0	0	0	0					
MV	BAEr	0	0	0	0	0	0	0	0	2.69 (9)	0
MV	BAOt	0	0	0	0	0	0	0	0.99 (12)	0.58 (19)	0
MV	BAWa	0	0	0	0	0	0	0	0	1.45 (12)	0
MV	BFLW	0	0	0	0	0	0	0	1.79 (11)	4.37 (6)	14.06 (3)
MV	BFOt	0	0	0	37.30 (2)	55.27 (1)	0	0	0	20.95 (1)	38.30 (1)
MV	BPGr	0	0	0	0	0	0	0	0	0	0
MV	BPMa	0	0	0	0	0	0	0	0	0	0
MV	BPno	0	0	0	0	0	0	0	14.84 (3)	0.73 (17)	0
MV	BPRi	0	0	0	0	0	0	0	0	0	0
MV	BPWo	0	0	0	0	0	0	0	0	0.19 (24)	0
MV	CBFO	0	0	0	0	0	0	0	0	0.43 (21)	0
MV	CBFR	17.04 (2)	23.19 (2)	28.01 (2)	0	0	16.02 (3)	34.75 (2)	15.88 (2)	10.25 (4)	12.83 (4)
MV	CFIB	0	0	0	0	0	0	0	4.89 (7)	9.44 (5)	10.07 (5)
MV	CFLW	0	0	0	0	0	0	0	0	0	0
MV	CFNa	0	0	0	0	0	0	0	0	0.24 (23)	0
MV	CFWi	0	0	0	0	0	0	0	0	0.19 (25)	0
MV	ChDe	0	0	0	0	0	2.77 (6)	7.42 (4)	6.72 (6)	1.99 (10)	0
MV	ChDV	0	0	0	0	0	0	0	0	0.02 (27)	0
MV	ChWi	2.12 (6)	2.79 (4)	0	0	0	38.52 (1)	35.16 (1)	27.97 (1)	20.21 (2)	24.74 (2)

		incl. TV					excl. TV				
Dataset	Variable	0	200	1000	2500	4000	0	200	1000	2500	4000
MV	ChWV	0	0	0	0	0	0	0	0	0.00 (28)	0
MV	CSFo	5.38 (4)	0	0	0	0	22.22 (2)	0	0	11.77 (3)	0
MV	FlDi	0	0	0	0	0	0	0	0	0	0
MV	FlVe	0	0	0	0	0	0	0	0	0.34 (22)	0
MV	InVe	0	0	0	0	0	0	0	0	0	0
MV	Plan	2.85 (5)	0	0	0	0	6.80 (5)	0	0	0.06 (26)	0
MV	RVRe	0	0	0	0	0	0	0	0	0	0
MV	RVSp	0	0	0	0	0	0	0	0	0.90 (14)	0
MV	RVTF	0	0	0	0	0	0	0	0	0.86 (15)	0
MV	SMaS	0	0	0	0	0	0	0	7.50 (5)	3.62 (8)	0
MV	SuDi	0	0	0	0	0	0	0	4.29 (8)	0.68 (18)	0
MV	SuHa	0	0	0	0	0	0	0	0	1.37 (13)	0
MV	SuMa	0	0	0	0	0	0	0	3.62 (9)	0.49 (20)	0
MV	SuSa	9.43 (3)	17.53 (3)	0	0	0	13.67 (4)	22.67 (3)	9.51 (4)	3.75 (7)	0
MV	SuSo	0	0	0	0	0	0	0	2.01 (10)	1.66 (11)	0
MV	SuWo	0	0	0	0	0	0	0	0	0.76 (16)	0
Salmario											
TV	DisM	45.63 (1)	28.72 (1)	20.87 (2)	25.82 (1)	16.75 (1)					
TV	SOSh	0	0	0	0	0					
TV	SOSt	0	0.05 (16)	0	0	0					
MV	BAEr	0	0.64 (12)	1.73 (9)	0	6.77 (7)	0	1.36 (12)	0	0	8.46 (6)
MV	BAOt	0	0	0	0	3.03 (13)	0	0	0	2.51 (10)	3.75 (12)
MV	BAWa	0	0	0	0	2.63 (14)	0	0	0	1.40 (12)	3.57 (13)
MV	BFLW	0	0	0	0	5.96 (8)	0	0	0	2.12 (11)	9.69 (4)
MV	BFOt	0	0	0	4.62 (8)	8.15 (5)	0	0	0	4.19 (8)	8.33 (7)
MV	BPGr	0	0	0	0	0	0	0	0	0	0
MV	BPMa	0	0	0	0	0	0	0	0	0	0
MV	BPno	0	0.15 (14)	0	0	0	0	0	0	0	0
MV	BPRi	0	0	0	0	3.64 (11)	0	0	0	0	5.94 (8)
MV	BPWo	0	0	0	0	0	0	0.97 (13)	0	0	0
MV	CBFO	0	0	0	0	8.76 (4)	0	0	0	0	9.41 (5)
MV	CBFR	0	5.13 (7)	0	5.95 (7)	0	0	7.61 (7)	0	10.07 (5)	0
MV	CFIB	0	0	0	0	0	0	0	0	0	0
MV	CFLW	0	0	0	0	0	0	0	0	0	0
MV	CFNa	0	0	7.97 (7)	8.35 (6)	4.61 (9)	0	0	0	7.24 (6)	4.59 (10)
MV	CFWi	0	0	0	0	0	0	0	0	0	0
MV	ChDe	0	2.62 (10)	1.41 (10)	3.22 (10)	0	0	4.68 (9)	0	4.21 (7)	0
MV	ChDV	0	0	0	0	0	0	0	0	0	0

Dataset	Variable	incl. TV					excl. TV				
		0	200	1000	2500	4000	0	200	1000	2500	4000
MV	ChWi	24.96 (2)	17.20 (2)	15.85 (3)	12.41 (3)	13.57 (2)	40.87 (1)	21.25 (1)	25.93 (2)	17.05 (2)	12.67 (2)
MV	ChWV	0	0	0	0	0	0	0	0	0	0
MV	CSFo	0	0	0	0	0	0	0	0	0	0
MV	FlDi	0	0	0	0	0	0	0	0	0	0
MV	FlVe	0	0	0	0	0	0	0	0	0	0
MV	InVe	0	0	0	0	0	0	0	0	0	0
MV	Plan	0	0	0	0	0	0	0	0	0	0
MV	RVRe	0	0	0	0	0	0	0	0	0	0
MV	RVSp	0	0.47 (13)	8.87 (5)	0	0	0	2.38 (10)	0	0	0
MV	RVTF	0	0.12 (15)	0	0	0	0	0	0	0	0
MV	SMaS	0	1.06 (11)	0	0	0	0	1.46 (11)	0	0	0
MV	SuDi	18.41 (3)	6.42 (6)	1.31 (11)	0	7.44 (6)	19.45 (3)	9.74 (4)	0	1.06 (13)	10.30 (3)
MV	SuHa	0	3.36 (9)	0	0	0	0	4.86 (8)	0	0	0
MV	SuMa	0	4.44 (8)	9.11 (4)	3.80 (9)	0	8.09 (5)	8.19 (6)	24.02 (3)	3.40 (9)	0
MV	SuSa	0	6.58 (5)	8.47 (6)	9.77 (5)	11.65 (3)	8.22 (4)	10.96 (3)	16.72 (4)	11.37 (4)	13.86 (1)
MV	SuSo	11.00 (4)	14.65 (3)	21.22 (1)	14.83 (2)	3.81 (10)	23.37 (2)	17.10 (2)	33.33 (1)	22.33 (1)	5.29 (9)
MV	SuWo	0	8.39 (4)	3.19 (8)	11.22 (4)	3.23 (12)	0	9.44 (5)	0	13.05 (3)	4.13 (11)
Tincinca											
TV	DisM	23.77 (2)	0	8.86 (3)	16.26 (3)	0					
TV	SOSh	0	0	0.20 (21)	0	0					
TV	SOSt	0	0	0.00 (23)	0	0					
MV	BAEr	0	0	2.64 (10)	0	0	0	0	2.69 (10)	0	0
MV	BAOt	0	0	42.38 (1)	17.35 (2)	48.28 (2)	0	0	49.24 (1)	0	48.34 (2)
MV	BAWa	0	0	1.39 (14)	0	0	0	0	1.27 (15)	0	0
MV	BFLW	0	0	0.72 (19)	0	0	0	0	0.96 (17)	0	0
MV	BFOt	0	0	2.52 (11)	0	0	0	0	2.25 (12)	0	0
MV	BPGr	0	0	0	0	0	0	0	0	0	0
MV	BPMa	0	0	0	0	0	0	0	0	0	0
MV	BPno	0	0	0.85 (18)	0	0	0	0	0	0	0
MV	BPRi	0	0	0	0	0	0	0	0	0	0
MV	BPWo	0	0	0.05 (22)	0	0	0	0	0	0	0
MV	CBFO	0	0	0	0	0	0	0	0	0	0
MV	CBFR	0	0	1.06 (16)	0	0	0	0	0	0	0
MV	CFIB	0	0	1.80 (13)	0	0	0	0	2.52 (11)	0	0
MV	CFLW	0	0	0	0	0	0	0	0	0	0
MV	CFNa	0	0	0	6.35 (6)	0	0	0	0	0	0
MV	CFWi	0	0	0	0	0	0	0	0	0	0
MV	ChDe	0	0	5.65 (4)	0	0	0	0	7.37 (2)	0	0

		incl. TV					excl. TV				
Dataset	Variable	0	200	1000	2500	4000	0	200	1000	2500	4000
MV	ChDV	0	0	0	0	0	0	0	0	0	0
MV	ChWi	3.87 (6)	0	3.44 (6)	0	0	0	0	3.75 (6)	0	0
MV	ChWV	0	0	0	0	0	0	0	0	0	0
MV	CSFo	10.39 (4)	0	0	0	0	19.96 (3)	0	0	0	0
MV	FlDi	0	0	0	0	0	0	0	0	0	0
MV	FlVe	0	0	0	0	0	0	0	0	0	0
MV	InVe	0	0	0	0	0	0	0	0	0	0
MV	Plan	9.62 (5)	0	0	0	0	13.95 (4)	0	0	0	0
MV	RVRe	0	0	0	0	0	0	0	0	0	0
MV	RVSp	0	0	0.68 (20)	0	0	0	0	1.30 (14)	0	0
MV	RVTF	0	0	3.40 (7)	34.64 (1)	0	0	0	3.18 (7)	0	0
MV	SMaS	0	0	1.01 (17)	0	0	0	0	1.18 (16)	0	0
MV	SuDi	0	0	2.32 (12)	0	0	0	0	2.78 (9)	0	0
MV	SuHa	0	0	2.94 (9)	0	0	0	0	3.14 (8)	0	0
MV	SuMa	0	0	3.30 (8)	15.08 (4)	51.72 (1)	0	0	3.97 (5)	0	51.66 (1)
MV	SuSa	20.51 (3)	51.57 (1)	4.05 (5)	0	0	29.44 (2)	52.14 (1)	5.73 (4)	0	0
MV	SuSo	31.84 (1)	48.43 (2)	9.49 (2)	10.32 (5)	0	36.65 (1)	47.86 (2)	7.32 (3)	0	0
MV	SuWo	0	0	1.25 (15)	0	0	0	0	1.35 (13)	0	0
