# Supplementary material for: Spatial Scaling of Environmental Variables Improves Species-Habitat Models of Fishes in a Small, Sand-Bed Lowland River
Source: PLoS One. 2015 Nov 16;10(11):e0142813. doi: 10.1371/journal.pone.0142813 (PMC4646645; doi:10.1371/journal.pone.0142813)
Supplement: S2 Table — (PDF) [file pone.0142813.s003.pdf]

S2 Table. Relative contribution of each predictor across five focal distance classes using assessed hydromorphological variables (AV) and with/without topological variables (TV). Descriptive information about predictor variables and corresponding values are provided in Table 2. Relative contribution (%) measured as the number of times a variable is used for splitting, weighted by the squared improvement at each split and averaged over all trees (Elith et al., 2008). Values in parenthesis indicate importance rank of each variable.

		incl. TV					excl. TV				
Dataset	Variable	0	200	1000	2500	4000	0	200	1000	2500	4000
Anguilla											
TV	DisM	18.93 (2)	9.58 (4)	0	0	13.50 (3)					
TV	SOSh	0	0	0	0	0					
TV	SOSt	19.18 (1)	12.67 (2)	26.99 (2)	0	0					
AV	FU-BaP	0	0	0	0	3.01 (8)	0	0	0	0	0
AV	FU-BeF	0	0	0	0	0	0	0	0	0	0
AV	FU-BFe	0	0	0	0	0	0	0	0	0	0
AV	FU-ChD	2.91 (10)	2.77 (10)	0	0	0.91 (10)	4.48 (8)	5.87 (9)	0	0	0
AV	FU-CSD	0	0	0	27.75 (3)	38.39 (1)	3.34 (9)	6.33 (8)	29.69 (2)	39.53 (2)	64.70 (1)
AV	FU-CSF	18.37 (3)	7.00 (6)	0	0	4.48 (6)	18.69 (3)	7.74 (6)	0	0	0
AV	FU-CSW	3.56 (8)	5.33 (8)	0	42.79 (1)	19.20 (2)	11.29 (4)	9.36 (4)	0	60.47 (1)	35.30 (2)
AV	FU-FPl	10.45 (5)	31.65 (1)	73.01 (1)	29.46 (2)	2.82 (9)	23.00 (1)	32.54 (1)	70.31 (1)	0	0
AV	FU-LPr	0	0	0	0	6.69 (5)	0	0	0	0	0
AV	FU-Pla	13.16 (4)	4.96 (9)	0	0	0	19.76 (2)	9.34 (5)	0	0	0
AV	FU-RBS	3.09 (9)	10.67 (3)	0	0	7.82 (4)	5.30 (7)	11.94 (2)	0	0	0
AV	FU-RVe	5.73 (6)	8.54 (5)	0	0	0	8.48 (5)	9.79 (3)	0	0	0
AV	FU-Sub	4.61 (7)	6.83 (7)	0	0	3.17 (7)	5.66 (6)	7.08 (7)	0	0	0
Cobienia											
TV	DisM	31.04 (1)	0	0	0	0					
TV	SOSh	18.11 (2)	25.00 (2)	0	0	0					
TV	SOSt	8.56 (6)	22.40 (3)	8.90 (4)	15.94 (4)	14.09 (4)					
AV	FU-BaP	0	0	0	0	0	0	0	0	9.97 (5)	0
AV	FU-BeF	0	0	0	0	0	0	0	0	0	0
AV	FU-BFe	0	0	25.50 (3)	0	0	0	0	26.83 (3)	0	0
AV	FU-ChD	0	0	0	0	0	0	0	0	0	0
AV	FU-CSD	0	0	31.69 (2)	20.72 (3)	28.32 (2)	32.81 (2)	48.87 (2)	40.41 (1)	29.04 (1)	40.48 (1)
AV	FU-CSF	0	0	0	0	0	0	0	0	10.96 (4)	0
AV	FU-CSW	0	0	0	29.88 (2)	34.10 (1)	0	0	0	18.38 (3)	33.66 (2)
AV	FU-FPl	0	0	0	0	0	0	0	0	0	0
AV	FU-LPr	0	0	0	0	0	0	0	0	6.32 (6)	0
AV	FU-Pla	10.59 (5)	0	0	0	0	0	0	0	0	0
AV	FU-RBS	0	0	0	0	0	0	0	0	0	0
AV	FU-RVe	16.55 (3)	0	0	0	0	37.66 (1)	0	0	0	0

		incl. TV					excl. TV				
Dataset	Variable	0	200	1000	2500	4000	0	200	1000	2500	4000
AV	FU-Sub	15.16 (4)	52.60 (1)	33.90 (1)	33.46 (1)	23.48 (3)	29.53 (3)	51.13 (1)	32.76 (2)	25.33 (2)	25.86 (3)
Gastatus											
TV	DisM	68.66 (1)	36.24 (2)	0	0	0					
TV	SOSh	0	0	0	0	0					
TV	SOSt	31.34 (2)	24.80 (3)	0	0	0					
AV	FU-BaP	0	0	0	0	0	0	0.61 (12)	0	0	0
AV	FU-BeF	0	0	0	0	0	0	0	0	0	0
AV	FU-BFe	0	0	0	0	0	0	2.49 (10)	0	0	0
AV	FU-ChD	0	0	0	0	0	0	9.69 (4)	0	0	0
AV	FU-CSD	0	0	30.44 (2)	46.74 (2)	67.89 (1)	0	7.28 (6)	21.04 (2)	46.91 (2)	52.10 (1)
AV	FU-CSF	0	0	0	0	32.11 (2)	0	1.52 (11)	2.91 (6)	0	21.38 (3)
AV	FU-CSW	0	0	0	0	0	0	6.58 (8)	0	0	0
AV	FU-FPl	0	0	0	0	0	0	10.57 (3)	0	0	0
AV	FU-LPr	0	38.96 (1)	69.56 (1)	53.26 (1)	0	0	20.60 (1)	36.27 (1)	53.09 (1)	26.53 (2)
AV	FU-Pla	0	0	0	0	0	0	6.81 (7)	9.76 (5)	0	0
AV	FU-RBS	0	0	0	0	0	0	7.52 (5)	20.13 (3)	0	0
AV	FU-RVe	0	0	0	0	0	0	6.49 (9)	0	0	0
AV	FU-Sub	0	0	0	0	0	0	19.83 (2)	9.88 (4)	0	0
Gobiobio											
TV	DisM	31.72 (2)	37.52 (2)	6.44 (8)	0	0					
TV	SOSh	0	0	0	0	0					
TV	SOSt	68.28 (1)	62.48 (1)	10.37 (4)	0	3.07 (4)					
AV	FU-BaP	0	0	0	0	0	0	0	0	0	0
AV	FU-BeF	0	0	0	0	0	0	0	0	0	0
AV	FU-BFe	0	0	0	0	0	0	0	0	0	0
AV	FU-ChD	0	0	14.42 (2)	0	0	28.04 (2)	28.68 (3)	17.33 (3)	0	0
AV	FU-CSD	0	0	31.11 (1)	93.87 (1)	89.85 (1)	71.96 (1)	36.19 (1)	32.93 (1)	95.56 (1)	94.93 (1)
AV	FU-CSF	0	0	0	0	0	0	0	0	0	0
AV	FU-CSW	0	0	0	0	3.60 (2)	0	35.14 (2)	0	0	0
AV	FU-FPl	0	0	0	0	0	0	0	9.69 (5)	0	0
AV	FU-LPr	0	0	12.29 (3)	0	0	0	0	17.35 (2)	0	0
AV	FU-Pla	0	0	8.62 (6)	6.13 (2)	3.48 (3)	0	0	9.23 (6)	4.44 (2)	5.07 (2)
AV	FU-RBS	0	0	6.78 (7)	0	0	0	0	0	0	0
AV	FU-RVe	0	0	9.97 (5)	0	0	0	0	13.47 (4)	0	0
AV	FU-Sub	0	0	0	0	0	0	0	0	0	0
Gymnrnua											
TV	DisM	36.14 (2)	40.71 (2)	18.44 (3)	18.32 (3)	38.14 (2)					
TV	SOSh	47.85 (1)	0	19.87 (2)	0	0					

		incl. TV					excl. TV				
Dataset	Variable	0	200	1000	2500	4000	0	200	1000	2500	4000
TV	SOSt	0	0	0	0	0					
AV	FU-BaP	0	0	0	0	0	0	0	0.74 (9)	0	0
AV	FU-BeF	0	0	0	0	0	0	0	0	0	0
AV	FU-BFe	0	0	0	0	0	0	0	0.88 (8)	0	0
AV	FU-ChD	0	0	0	0	0	0	0	0.41 (11)	0	0
AV	FU-CSD	9.07 (3)	59.29 (1)	27.62 (1)	33.45 (1)	61.86 (1)	76.12 (1)	60.20 (1)	66.97 (1)	51.79 (1)	69.92 (1)
AV	FU-CSF	0	0	0.32 (11)	0	0	0	0	0.55 (10)	0	0
AV	FU-CSW	0	0	2.60 (9)	2.03 (8)	0	0	0	2.00 (7)	0	0
AV	FU-FPl	0	0	0.80 (10)	0	0	0	0	0.26 (12)	0	0
AV	FU-LPr	0	0	7.70 (5)	3.23 (6)	0	23.88 (2)	39.80 (2)	8.71 (3)	0	0
AV	FU-Pla	0	0	12.64 (4)	2.17 (7)	0	0	0	9.14 (2)	0	30.08 (2)
AV	FU-RBS	0	0	2.80 (7)	29.99 (2)	0	0	0	3.06 (5)	48.21 (2)	0
AV	FU-RVe	6.94 (4)	0	4.53 (6)	4.91 (5)	0	0	0	4.33 (4)	0	0
AV	FU-Sub	0	0	2.68 (8)	5.91 (4)	0	0	0	2.93 (6)	0	0
Leucscus											
TV	DisM	45.29 (1)	25.04 (1)	19.66 (1)	20.67 (2)	27.65 (2)					
TV	SOSh	0	1.15 (12)	1.31 (14)	0	0					
TV	SOSt	10.73 (4)	4.25 (9)	3.14 (10)	0	0					
AV	FU-BaP	0	0	1.07 (15)	0	0	0	0.18 (12)	0	0	0
AV	FU-BeF	0	0	0	0	0	0	0	0	0	0
AV	FU-BFe	0	0	2.75 (11)	9.90 (5)	18.98 (4)	0	0.35 (11)	2.05 (10)	8.96 (5)	25.15 (3)
AV	FU-ChD	6.82 (5)	11.41 (4)	3.79 (7)	19.59 (3)	30.76 (1)	32.69 (2)	20.62 (1)	7.64 (5)	25.83 (2)	43.93 (1)
AV	FU-CSD	0	0	3.75 (8)	6.82 (6)	0	0	1.02 (10)	5.79 (7)	8.91 (6)	0
AV	FU-CSF	20.63 (2)	15.00 (2)	17.72 (2)	22.72 (1)	22.61 (3)	36.75 (1)	19.43 (2)	23.59 (1)	27.05 (1)	30.91 (2)
AV	FU-CSW	0	7.18 (5)	4.98 (6)	6.13 (7)	0	0	9.89 (5)	6.88 (6)	9.89 (4)	0
AV	FU-FPl	0	5.92 (7)	2.02 (13)	0	0	0	12.70 (4)	1.50 (11)	0	0
AV	FU-LPr	0	5.03 (8)	7.72 (5)	0	0	0	5.96 (7)	9.09 (4)	0	0
AV	FU-Pla	16.53 (3)	6.37 (6)	16.79 (3)	0	0	30.56 (3)	7.07 (6)	22.10 (2)	0	0
AV	FU-RBS	0	3.02 (11)	3.30 (9)	0	0	0	3.10 (9)	2.39 (9)	0	0
AV	FU-RVe	0	3.74 (10)	2.47 (12)	0	0	0	4.08 (8)	3.59 (8)	0	0
AV	FU-Sub	0	11.90 (3)	9.52 (4)	14.18 (4)	0	0	15.59 (3)	15.37 (3)	19.36 (3)	0
Percilis											
TV	DisM	0	0	0	0	0					
TV	SOSh	72.55 (1)	74.23 (1)	58.33 (1)	0	15.71 (3)					
TV	SOSt	27.45 (2)	25.77 (2)	18.23 (3)	8.35 (3)	8.76 (6)					
AV	FU-BaP	0	0	0	0	0	0	0	0	0	0
AV	FU-BeF	0	0	0	0	0	0	0	0	0	0
AV	FU-BFe	0	0	0	0	0	0	0	0	0	0

		incl. TV					excl. TV				
Dataset	Variable	0	200	1000	2500	4000	0	200	1000	2500	4000
AV	FU-ChD	0	0	0	0	0	28.45 (1)	18.08 (3)	28.50 (2)	0	0
AV	FU-CSD	0	0	23.44 (2)	63.91 (1)	34.82 (1)	27.30 (2)	39.94 (1)	71.50 (1)	72.60 (1)	77.87 (1)
AV	FU-CSF	0	0	0	0	18.82 (2)	7.10 (5)	0	0	0	0
AV	FU-CSW	0	0	0	0	0	0	15.87 (4)	0	0	0
AV	FU-FPl	0	0	0	0	0	15.47 (4)	26.11 (2)	0	0	0
AV	FU-LPr	0	0	0	0	11.11 (4)	0	0	0	0	0
AV	FU-Pla	0	0	0	27.74 (2)	10.78 (5)	21.68 (3)	0	0	27.40 (2)	22.13 (2)
AV	FU-RBS	0	0	0	0	0	0	0	0	0	0
AV	FU-RVe	0	0	0	0	0	0	0	0	0	0
AV	FU-Sub	0	0	0	0	0	0	0	0	0	0
Phoxinus											
TV	DisM	34.47 (1)	21.87 (1)	19.65 (1)	17.21 (3)	12.75 (4)					
TV	SOSh	25.79 (2)	9.82 (4)	11.16 (4)	9.07 (5)	2.00 (10)					
TV	SOSt	3.37 (8)	1.33 (12)	0	0	0					
AV	FU-BaP	0	0	1.02 (13)	0	2.78 (8)	0.33 (12)	0	0	0	6.43 (5)
AV	FU-BeF	0	0	0	0	0	0	0	0	0	0
AV	FU-BFe	0	0.68 (14)	0	0	0	5.00 (8)	0	0	0	0
AV	FU-ChD	9.33 (3)	20.17 (2)	11.46 (3)	8.79 (6)	12.81 (3)	27.42 (1)	28.49 (1)	19.26 (2)	12.94 (4)	13.87 (3)
AV	FU-CSD	1.29 (11)	7.07 (5)	15.47 (2)	20.59 (2)	28.98 (1)	13.50 (2)	12.61 (3)	20.01 (1)	30.16 (2)	31.01 (1)
AV	FU-CSF	5.76 (4)	3.07 (11)	3.73 (9)	0	0	9.01 (4)	4.64 (7)	5.46 (9)	0	3.72 (9)
AV	FU-CSW	4.82 (6)	6.76 (6)	6.26 (7)	0	1.82 (11)	8.66 (5)	10.01 (5)	9.90 (4)	0	2.29 (10)
AV	FU-FPl	0	6.03 (7)	3.01 (11)	6.52 (7)	6.23 (6)	7.39 (6)	10.76 (4)	6.60 (6)	9.77 (5)	6.93 (4)
AV	FU-LPr	5.38 (5)	4.51 (8)	4.63 (8)	14.05 (4)	6.87 (5)	2.54 (11)	3.86 (9)	3.73 (10)	15.28 (3)	5.36 (6)
AV	FU-Pla	3.25 (9)	3.17 (10)	11.09 (5)	23.76 (1)	19.27 (2)	4.37 (9)	4.19 (8)	14.81 (3)	31.84 (1)	21.89 (2)
AV	FU-RBS	2.80 (10)	10.67 (3)	6.59 (6)	0	0	10.98 (3)	15.71 (2)	8.85 (5)	0	0
AV	FU-RVe	3.75 (7)	3.79 (9)	2.48 (12)	0	3.75 (7)	6.98 (7)	7.20 (6)	5.70 (7)	0	3.99 (8)
AV	FU-Sub	0	1.06 (13)	3.44 (10)	0	2.75 (9)	3.82 (10)	2.53 (10)	5.67 (8)	0	4.52 (7)
Pungtius											
TV	DisM	13.37 (3)	0	9.70 (7)	0	0					
TV	SOSh	0.87 (9)	0	0	0	0					
TV	SOSt	0.20 (13)	0	0	0	0					
AV	FU-BaP	0	0	0	0	0	0	0	0	0	0
AV	FU-BeF	0	0	0	0	0	0	0	0	0	0
AV	FU-BFe	0	0	3.46 (8)	26.57 (2)	19.87 (4)	0	0	4.73 (6)	0	14.16 (5)
AV	FU-ChD	34.38 (1)	44.26 (1)	12.51 (3)	0	0	52.52 (1)	39.74 (1)	14.46 (5)	0	6.53 (6)
AV	FU-CSD	0.63 (11)	0	0	0	0	0	0	0	0	0
AV	FU-CSF	4.14 (7)	0	18.21 (2)	12.15 (3)	27.05 (2)	7.34 (4)	0	22.07 (2)	23.97 (2)	21.22 (2)
AV	FU-CSW	0.57 (12)	0	10.97 (4)	53.87 (1)	23.09 (3)	0	0	15.47 (4)	76.03 (1)	20.50 (3)

		incl. TV					excl. TV				
Dataset	Variable	0	200	1000	2500	4000	0	200	1000	2500	4000
AV	FU-FPl	11.09 (4)	0	0	0	0	0	0	0	0	14.44 (4)
AV	FU-LPr	7.98 (5)	25.08 (3)	10.55 (5)	7.41 (4)	30.00 (1)	0	29.03 (3)	17.55 (3)	0	23.15 (1)
AV	FU-Pla	17.58 (2)	30.65 (2)	24.75 (1)	0	0	27.80 (2)	31.23 (2)	25.72 (1)	0	0
AV	FU-RBS	5.66 (6)	0	9.84 (6)	0	0	12.35 (3)	0	0	0	0
AV	FU-RVe	2.69 (8)	0	0	0	0	0	0	0	0	0
AV	FU-Sub	0.83 (10)	0	0	0	0	0	0	0	0	0
Rutiilus											
TV	DisM	0	0	0	0	0					
TV	SOSh	0	0	0	0	0					
TV	SOSt	75.41 (1)	33.64 (2)	50.82 (1)	50.05 (1)	67.85 (1)					
AV	FU-BaP	0	0	0	0	0	0	0	0	0	0
AV	FU-BeF	0	0	0	0	0	0	0	0	0	0
AV	FU-BFe	0	0	0	0	0	0	0	0	0	0
AV	FU-ChD	0	0	0	0	0	19.16 (4)	0	0	0	0
AV	FU-CSD	0	0	0	49.95 (2)	0	24.82 (2)	0	41.58 (2)	70.64 (1)	62.66 (1)
AV	FU-CSF	0	0	0	0	0	0	0	0	0	0
AV	FU-CSW	24.59 (2)	43.73 (1)	49.18 (2)	0	0	35.95 (1)	64.85 (1)	58.42 (1)	29.36 (2)	0
AV	FU-FPl	0	22.63 (3)	0	0	0	0	35.15 (2)	0	0	0
AV	FU-LPr	0	0	0	0	32.15 (2)	0	0	0	0	37.34 (2)
AV	FU-Pla	0	0	0	0	0	0	0	0	0	0
AV	FU-RBS	0	0	0	0	0	0	0	0	0	0
AV	FU-RVe	0	0	0	0	0	0	0	0	0	0
AV	FU-Sub	0	0	0	0	0	20.07 (3)	0	0	0	0
Salmalar											
TV	DisM	0	0	0	0	0					
TV	SOSh	83.87 (1)	67.78 (1)	65.06 (1)	49.52 (1)	9.15 (4)					
TV	SOSt	0	0	0	0	0					
AV	FU-BaP	0	0	2.47 (5)	0	0	0	0	9.20 (3)	0.32 (10)	0
AV	FU-BeF	0	0	0	0	0	0	0	0	0	0
AV	FU-BFe	0	0	7.94 (3)	18.50 (3)	51.17 (1)	0	0	0	15.80 (3)	69.68 (1)
AV	FU-ChD	16.13 (2)	11.26 (3)	17.53 (2)	31.98 (2)	11.37 (3)	46.16 (1)	41.63 (1)	61.42 (1)	26.37 (2)	30.32 (2)
AV	FU-CSD	0	0	1.83 (6)	0	7.07 (5)	24.26 (2)	28.61 (3)	21.86 (2)	12.85 (4)	0
AV	FU-CSF	0	0	0	0	14.76 (2)	9.75 (3)	0	0	3.18 (6)	0
AV	FU-CSW	0	0	0	0	0	2.99 (8)	0	0	0	0
AV	FU-FPl	0	0	0	0	1.18 (8)	0	0	0	0.12 (11)	0
AV	FU-LPr	0	0	0	0	0	1.68 (9)	0	0	1.15 (9)	0
AV	FU-Pla	0	20.96 (2)	5.18 (4)	0	1.92 (7)	3.91 (5)	29.76 (2)	7.53 (4)	29.55 (1)	0
AV	FU-RBS	0	0	0	0	3.40 (6)	3.33 (7)	0	0	2.40 (7)	0

		incl. TV					excl. TV				
Dataset	Variable	0	200	1000	2500	4000	0	200	1000	2500	4000
AV	FU-RVe	0	0	0	0	0	3.34 (6)	0	0	1.54 (8)	0
AV	FU-Sub	0	0	0	0	0	4.58 (4)	0	0	6.71 (5)	0
Salmario											
TV	DisM	85.62 (1)	60.28 (1)	56.88 (1)	53.61 (1)	50.94 (1)					
TV	SOSh	0	0	0	0	0					
TV	SOSt	10.65 (2)	5.16 (6)	0	0	0					
AV	FU-BaP	0	0	0	2.01 (10)	1.60 (11)	0	0	0	3.32 (9)	5.81 (7)
AV	FU-BeF	0	0	0	0	0	0	0	0	0	0
AV	FU-BFe	0	0	0	3.52 (7)	2.43 (9)	0	0	0	5.02 (8)	5.66 (8)
AV	FU-ChD	3.72 (3)	2.71 (7)	0	0.51 (13)	0.64 (12)	63.44 (1)	20.93 (3)	0	1.65 (11)	2.63 (11)
AV	FU-CSD	0	0	16.72 (3)	8.74 (2)	8.73 (3)	0	0	39.07 (1)	16.94 (2)	14.85 (2)
AV	FU-CSF	0	0	0	2.86 (9)	3.11 (8)	0	0	0	6.16 (6)	4.58 (9)
AV	FU-CSW	0	0	0	8.56 (3)	3.30 (7)	0	0	0	12.69 (4)	7.70 (6)
AV	FU-FPl	0	12.40 (2)	0	4.76 (5)	4.83 (5)	0	52.37 (1)	31.51 (2)	21.57 (1)	14.04 (4)
AV	FU-LPr	0	0	0	1.80 (12)	4.20 (6)	0	0	0	3.28 (10)	4.30 (10)
AV	FU-Pla	0	8.82 (3)	0	3.63 (6)	7.17 (4)	0	26.70 (2)	0	13.12 (3)	16.63 (1)
AV	FU-RBS	0	5.45 (4)	0	1.93 (11)	0	0	0	0	0	0
AV	FU-RVe	0	5.17 (5)	0	5.08 (4)	10.85 (2)	0	0	29.42 (3)	5.28 (7)	14.52 (3)
AV	FU-Sub	0	0	26.40 (2)	2.98 (8)	2.20 (10)	36.56 (2)	0	0	10.96 (5)	9.28 (5)
Tincinca											
TV	DisM	42.45 (1)	30.77 (1)	0	0	0					
TV	SOSh	0	0	0	0	0					
TV	SOSt	0	0	0	0	0					
AV	FU-BaP	0	0	0	0	0	0	0	0	0	0
AV	FU-BeF	0	0	0	0	0	0	0	0	0	0
AV	FU-BFe	0	0	24.99 (3)	0	22.14 (3)	0	0	23.82 (3)	0	23.38 (2)
AV	FU-ChD	15.90 (2)	4.71 (6)	0	26.78 (2)	23.12 (2)	23.84 (3)	0	0	25.49 (2)	22.67 (3)
AV	FU-CSD	0	0	0	0	0	0	0	0	0	0
AV	FU-CSF	11.82 (4)	6.37 (5)	41.63 (1)	73.22 (1)	54.74 (1)	25.81 (2)	0	40.75 (1)	74.51 (1)	53.95 (1)
AV	FU-CSW	11.08 (5)	26.88 (2)	0	0	0	20.22 (4)	51.68 (1)	0	0	0
AV	FU-FPl	0	0	0	0	0	0	0	0	0	0
AV	FU-LPr	3.68 (6)	9.58 (4)	0	0	0	0	0	0	0	0
AV	FU-Pla	15.06 (3)	21.68 (3)	33.38 (2)	0	0	30.13 (1)	48.32 (2)	35.43 (2)	0	0
AV	FU-RBS	0	0	0	0	0	0	0	0	0	0
AV	FU-RVe	0	0	0	0	0	0	0	0	0	0
AV	FU-Sub	0	0	0	0	0	0	0	0	0	0
